# Minimizing Sensor-Sample Distances in Scanning Nitrogen-Vacancy Magnetometry

**DOI:** 10.1021/acsnano.4c18460

**Published:** 2025-02-21

**Authors:** Zhewen Xu, Marius L. Palm, William Huxter, Konstantin Herb, John M. Abendroth, Karim Bouzehouane, Olivier Boulle, Mihai S. Gabor, Joseba Urrestarazu Larranaga, Andrea Morales, Jan Rhensius, Gabriel Puebla-Hellmann, Christian L. Degen

**Affiliations:** ‡ Department of Physics, ETH Zürich, Otto Stern Weg 1, 8093 Zürich, Switzerland; § QZabre AG, Neubrunnenstrasse 50, 8050 Zürich, Switzerland; ⊥ Laboratoire Albert Fert, CNRS, Thales, Université Paris-Saclay, 91767 Palaiseau, France; ∥ Université Grenoble Alpes, CNRS, CEA, SPINTEC, 38054 Grenoble, France; ¶ Technical University of Cluj-Napoca, Memorandumului 28, Cluj-Napoca 400347, Romania; # Quantum Center, ETH Zürich, 8093 Zürich, Switzerland

**Keywords:** diamond NV center, scanning probe microscopy, spatial resolution, magnetic imaging, capillary bridge, surface adsorbates, NMR spectroscopy

## Abstract

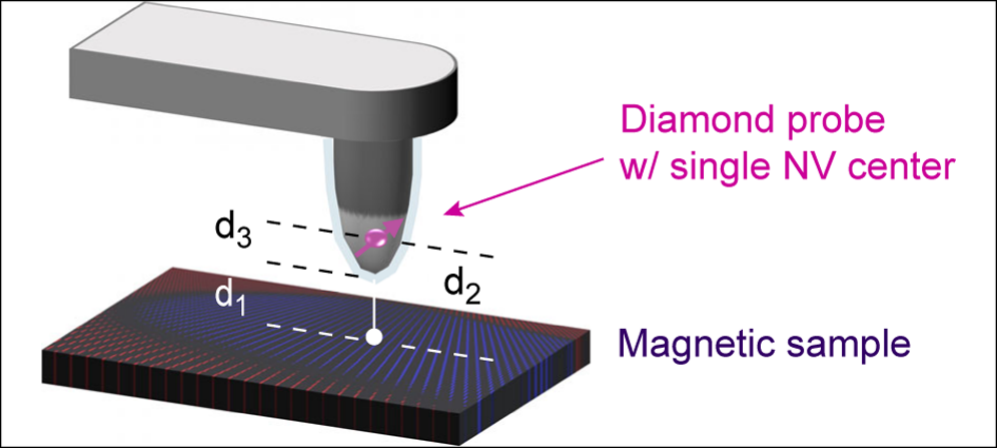

Scanning magnetometry with nitrogen-vacancy (NV) centers in diamond has led to significant advances in the sensitive imaging of magnetic systems. The spatial resolution of the technique, however, remains limited to tens to hundreds of nanometers, even for probes where NV centers are engineered within 10 nm from the tip apex. Here, we present a correlated investigation of the crucial parameters that determine the spatial resolution: the mechanical and magnetic stand-off distances, as well as the subsurface NV center depth in diamond. We study their contributions using mechanical approach curves, photoluminescence measurements, magnetometry scans, and nuclear magnetic resonance (NMR) spectroscopy of surface adsorbates. We first show that the stand-off distance is mainly limited by features on the surface of the diamond tip, hindering mechanical access. Next, we demonstrate that frequency-modulated (FM) atomic force microscopy feedback partially overcomes this issue, leading to closer and more consistent magnetic stand-off distances (26–87 nm) compared with the more common amplitude-modulated feedback (43–128 nm). FM operation thus permits improved magnetic imaging of sub-100-nm spin textures, shown for the spin cycloid in BiFeO_3_ and domain walls in a CoFeB synthetic antiferromagnet. Finally, by examining ^1^H and ^19^F NMR signals in soft contact with a polytetrafluoroethylene surface, we demonstrate a minimum NV-to-sample distance of 7.9 ± 0.4 nm.

Magnetic imaging techniques are indispensable tools in nanoscale research, with applications ranging from fundamental research in condensed matter and materials physics to metrology and device characterization in engineering disciplines. For example, magnetic force microscopy^1,2^ and Lorentz transmission electron microscopy^3,4^ are widely used to reveal domain patterns and spin textures, which are essential for understanding the energy scales and dynamics of magnetically ordered systems. Similar and complementary information is available from a range of other techniques, such as scanning superconducting quantum interference device microscopy,^5^ a number of scanning tunneling microscopies,^6−8^ and X-ray methods such as magnetic circular dichroism and magnetic linear dichroism with photoemission electron microscopy.^9^ While all techniques offer nanoscale or even atomic spatial resolution, each method has its specific requirements that limit applications to certain samples or operating conditions. For example, some techniques only accept samples as thin films, while others require atomically flat and conducting surfaces, an ultrahigh-vacuum (UHV) environment, or cryogenic operation. In addition, X-ray investigations rely on access to large-scale facilities where the measurement time is limited.

Scanning magnetometers based on nitrogen-vacancy centers (SNVMs) are an important recent addition to the set of nanoscale magnetic imaging instruments.^10−12^ SNVMs exploit a single defect spin in a diamond scanning tip as an atomic-size magnetic sensor, allowing for quantitative and sensitive stray field imaging at sub-100-nm spatial resolution. Such microscopes are tabletop instruments, operate under ambient conditions, and are compatible with a wide range of samples, including bulk and thin-film materials. Initially applied to study magnetic textures in ferromagnets,^13−16^ the technique has since been extended to antiferromagnets,^17−21^ multiferroics,^22−24^ two-dimensional ferromagnets,^25−27^ skyrmions,^28−30^ superconducting vortices,^31,32^ and nanoscale current distributions.^33,34^ While SNVM offers excellent sensitivity in real space imaging, the spatial resolution, typically tens of nanometers, is modest compared to related techniques like magnetic force microscopy (MFM) or scanning tunneling microscopy (STM).^35^ The limited spatial resolution hinders investigation of a range of interesting phenomena on the ∼10 nm length scale, such as the internal structure of magnetic domain walls,^36^ superlattice structures,^37^ or magnetic signatures related to wave effects of electrons.^38,39^

In SNVM, the spatial resolution is directly set by the vertical separation (stand-off) between the atomic-size magnetic sensor and the sample surface.^33^ Therefore, to attain a high spatial resolution, the stand-off must be reduced as much as possible. Early approaches based on grafting a diamond nanoparticle to a commercial atomic force microscopy (AFM) probe occasionally reported excellent stand-off distances (15–25 nm^33,40^). However, this approach is difficult to reproduce, and the quantum properties of NV centers in nanocrystals are generally poor.^42,43^ By contrast, state-of-the-art scanning probes fabricated by top-down lithography from single-crystalline diamond substrates^44,45^ offer excellent NV properties. However, stand-off distances (35–120 nm^22,46−54^) are generally larger and vary greatly. The reasons for such large stand-offs are unclear, as the nominal subsurface depth of NV centers is expected to be around 10 nm, based on the nitrogen implantation energy during NV synthesis. Possible explanations are tip topography caused by surface roughness or particle pickup, tip tilt relative to the sample, meniscus formation by surface adsorbates between the tip and sample, or a biased selection of deep NV centers in tip fabrication. Routinely achieving stand-off distances below 50 nm (ideally less than 10 nm) remains a fundamental and practical challenge.

In this work, we present a systematic study of stand-off distances in SNVM imaging. First, we characterize the critical vertical distance parameters—the mechanical stand-off (*d*_1_), the magnetic stand-off (*d*_2_), and the subsurface NV depth (*d*_3_) (cf. Figure 1a)—to form a detailed picture of the sensor-sample interaction. We show that there is considerable variation in magnetic stand-off between probes, likely due to a combination of inadvertent tip contamination and meniscus formation common to large-diameter AFM probes. By contrast, the stand-off contribution due to the subsurface depth of the NV center is minor. We further show that, by using frequency-modulation (FM) feedback for the AFM tip approach and optimizing control parameters, the magnetic stand-off can be lowered by ∼16 nm (median) compared to amplitude-modulation (AM) feedback. We demonstrate measurement improvement by imaging BiFeO_3_ and CoFeB magnetic test samples at stand-offs down to 24.3 ± 4.6 nm, as well as NMR detection of surface films in soft contact with a stand-off of 7.9 ± 0.4 nm.

**Figure 1:**
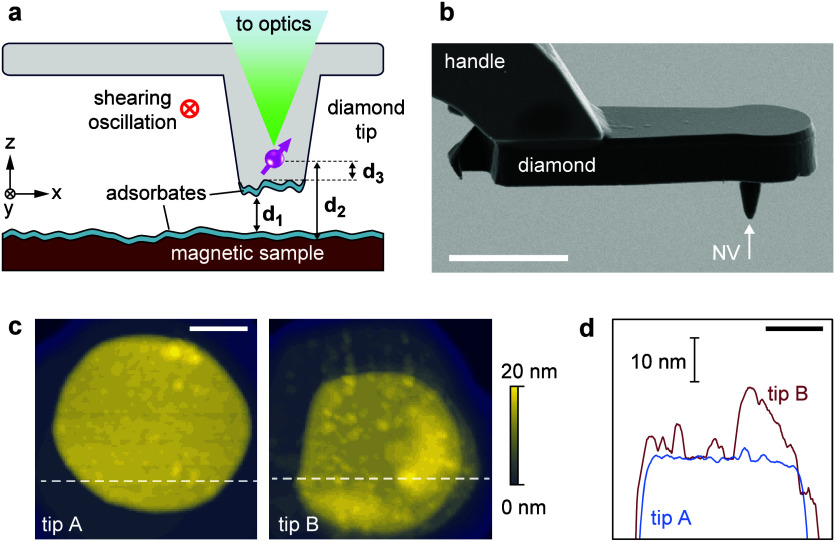
Scanning NV microscope and diamond tip. (a) Schematic of the scanning probe and surface, indicating the mechanical stand-off (*d*_1_), magnetic stand-off (*d*_2_), and subsurface depth of the NV center (*d*_3_). The diamond probe is oscillated along *y* (shear mode) using a tuning-fork actuator. A combination of optical and microwave excitation is used to control and read out the NV spin (pink). (b) Electron micrograph of a diamond scanning probe. The NV center is located at the tip apex (arrow). Scale bar, 10 μm. (c) AFM topography of the tip apex from two representative diamond probes with smooth (tip A) and rough (tip B) surfaces, respectively. (d) Line cuts along the dashed lines in part c. The scale bars in parts c and d are 200 nm.

## Results and Discussion

### Diamond NV Tips

Our diamond probes^55^ are fabricated from single-crystal diamond substrates using a series of e-beam lithography and dry etching steps.^45^ Each probe contains a single NV defect center approximately 10 nm from the tip apex formed by low-energy nitrogen implantation (7 keV^15^ N^+^) and subsequent annealing.^56^ Tips have a tapered geometry with top diameters between 350 and 400 nm,^45^ which protrude from a larger diamond piece that is glued, via a cantilever handle, to a quartz tuning fork anchored on a ceramic chip for easy handling and electrical contacting (Figure 1b). All measurements are performed with a commercial scanning NV magnetometer^55^ under ambient conditions (cf. Figure 1a and Methods).

Parts c and d of Figure 1 show AFM scans of the tip apex from two diamond probes. These tips are from a separate chip^45^ and have larger diameters (∼700 nm) compared to the scanning tips analyzed in the rest of this study. Although the tip apex is in general very smooth (tip A in Figure 1c), with an root-mean-square (rms) roughness below 1 nm, some tips (tip B) showed larger features with typical peak heights of 20–50 nm (see Supplementary Note 1 for statistics). These features are important because they hinder mechanical access during the tip approach and therefore increase the stand-off distance. The likely origin for features seen on tips of type B are residues from tip lithography, such as mask material or photoresist. The images in Figure 1c are taken on pristine tips; images acquired from scanning probes in reverse AFM mode before use (Supplementary Note 1) revealed similar features on the tip surface. As with any AFM imaging, tips tend to accumulate more material during use when scanning in close contact^57^ and under prolonged laser exposure^58^ even under UHV conditions.^59^

### Mechanical Approach Curves

**Figure 2:**
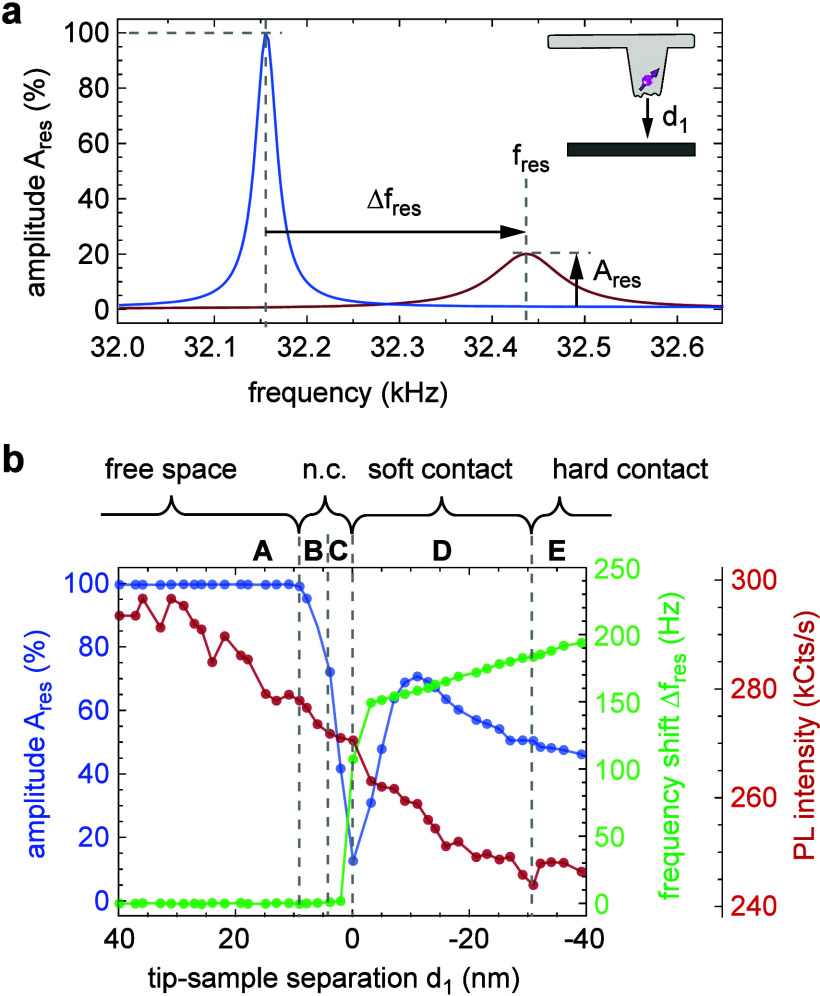
Tuning-fork control and approach curves. (a) Tuning-fork resonance curves in free space (blue) and in hard contact (red). *A*_res_ is the resonant amplitude and Δ*f*_res_ is the frequency shift, given relative to the free-space (*d*_1_ → *∞*) value. (b) Approach curves plotting *A*_res_ (blue), Δ*f*_res_ (green), and the PL intensity (red) as a function of *d*_1_. The contact point *d*_1_ = 0 is defined by the minimum in *A*_res_. A–E identify different interaction regimes discussed in the text. The data shown are for diamond tip NV14 and a Si/SiO_2_ sample surface. n.c. = noncontact.

We begin our study by quantifying the mechanical stand-off distance, *d*_1_, corresponding to the physical separation between the lowest point of the diamond probe tip and the highest point of the sample surface (Figure 1a). For this purpose, we record the resonant amplitude (*A*_res_) and frequency (*f*_res_) of the tuning fork (Figure 2a) while slowly approaching the sample surface. By plotting *A*_res_ and *f*_res_ as a function of *d*_1_, we obtain the approach curves shown in Figure 2b. In addition, we monitor the photoluminescence (PL) intensity of the NV center under constant green laser illumination. Because large-diameter tips operated in shear mode behave differently from standard tapping mode AFM tips^60^ and because an understanding of the approach curves is crucial for choosing the set point and minimizing *d*_1_, we now discuss Figure 2b in some detail.

In the free-space regime A, no mechanical tip–sample interaction is detected. *A*_res_ and *f*_res_ take their free-space values. However, a reduction in the PL intensity is already visible. The reduction in PL is mainly due to an optical cavity forming between the parallel tip and sample surfaces.^61−63^ In region B, the amplitude *A*_res_ starts decreasing due to dissipative tip–sample interactions,^64−66^ while *f*_res_ is largely unaffected, indicating that dissipative tip–sample interactions occur prior to conservative tip–sample interactions. In region C, conservative tip–sample interactions start contributing, leading to a rise in the *f*_res_ value. Crossing from region C into region D, *A*_res_ assumes a sharp minimum. Because this minimum is a well-defined feature, we use it to define the “contact point” (*d*_1_ = 0) in the approach curve.^65,67^ The contact point reflects a soft contact, and the tip can be approached further toward the sample. In region D, the amplitude increases again and eventually reaches a local maximum. This maximum is attributed to adsorbates, such as water, in ambient conditions, filling the gap between the diamond tip and the sample surface. As a consequence, the cantilever experiences an increasing shear force and begins to bend elastically.^66^ Finally, in region E, the PL signal saturates and *A*_res_ and *f*_res_ vary slowly with the *z* position, indicating that a hard contact has been made.

To control the tip–sample distance, we feedback on either the amplitude or frequency signal (Methods). In amplitude-modulated (AM) feedback mode, the tuning fork is driven at a fixed frequency, and the oscillation amplitude is held at a constant set point by adjusting the *z* position using a proportional–integral (PI) controller. The set point may be arbitrarily chosen, typically between 20 and 90% of the free amplitude (cf. Figure 2a). In frequency-modulated (FM) feedback mode, the tuning fork is driven at resonance with the help of a phase-locked loop (PLL), and the frequency shift (typically 5–200 Hz) is held at a constant set point.

We find that the FM feedback has several key advantages over the AM feedback: first, because the *A*_res_ signal reacts earlier than the *f*_res_ signal, the AM set point is typically farther from the sample than the FM set point. Further, the nonmonotonic behavior of *A*_res_, especially the dip and local maximum in regions C and D, tends to make the feedback unstable. As a consequence, a set point in regions B and C must be chosen in the AM mode. By contrast, *f*_res_ increases monotonically with decreasing *d*_1_ even deep into the soft-contact regime, allowing for a much closer approach, combined with more robust and reproducible feedback in the FM mode. Therefore, with careful tuning of *f*_set_, the FM mode is expected to allow for substantially smaller and more consistent stand-off distances compared to the AM mode.

### Magnetic Stand-off Distance

Next, we investigate the magnetic stand-off, *d*_2_, given by the vertical distance between the NV center and the top surface of the magnetic sample (Figure 1a). Our calibration sample is an out-of-plane (OOP) magnetized ferromagnetic Co film of 1.6 nm thickness that is lithographically patterned into a 2-μm-wide stripe.^68,69^ To determine the magnetic stand-off, we take magnetometry line scans across the stripe and fit the data to an analytical function describing the stray field from a uniformly magnetized OOP stripe (Methods).^46,47^

**Figure 3:**
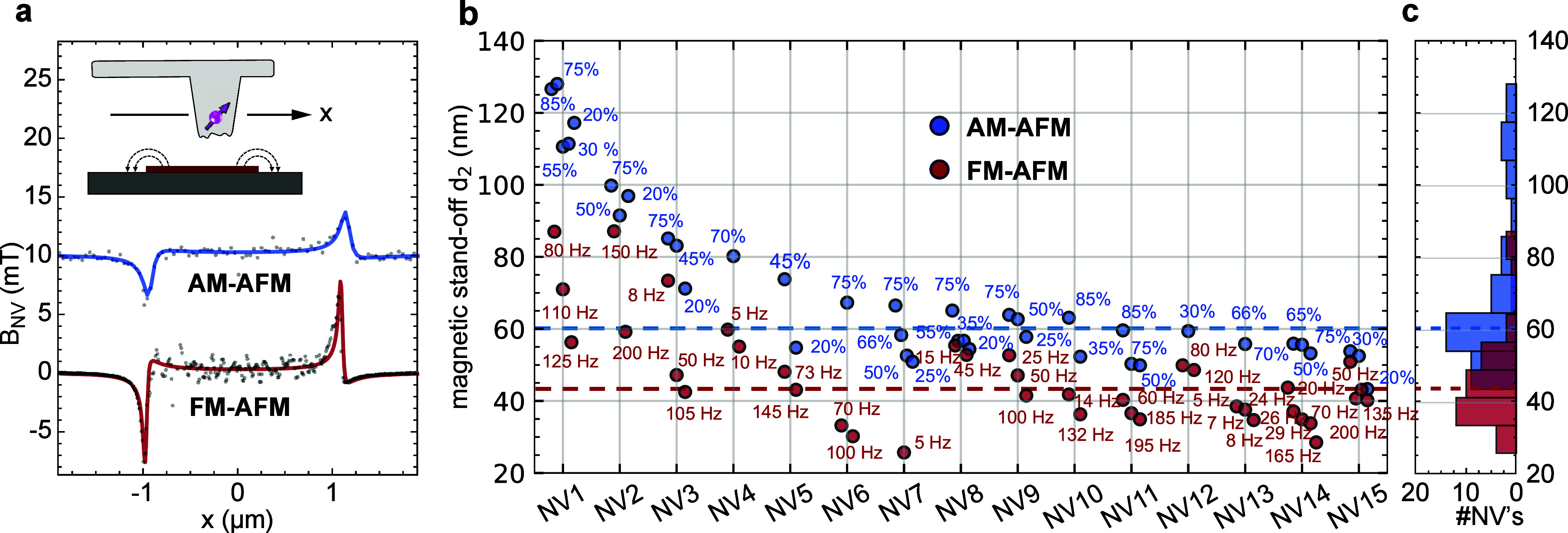
Magnetic stand-off distance. (a) Stray field scans for NV7 obtained by scanning the tip across an OOP magnetized ferromagnetic stripe (inset). The upper trace (blue, offset by 10 mT for clarity) is recorded in the AM mode and the lower trace (red) in the FM mode. Dots show the data, and solid lines are the analytical fits to the stray field profile (Methods). (b) Scatter plot of magnetic stand-off distances *d*_2_ from diamond probes (NV1–NV15), totaling 77 scans. NV indices are ordered according their AM-AFM stand-off. Blue and red numbers indicate set-point parameters (see the text). (c) Histogram of the data shown in part b. The dashed horizontal lines are median values.

Figure 3a depicts measured stray field profiles and model fits for one representative tip (NV7) acquired in the AM (blue) and FM (red) feedback modes. The FM feedback yields a sharper field profile and an ∼2 times larger absolute stray field, as expected for a scan in closer proximity. For this example, the fitted magnetic stand-off distances are 58.3 ± 1.5 nm (AM) and 25.7 ± 1.4 nm (FM). This result is consistent with the ∝*d*_2_^–1^ scaling of the stray field peak from a step edge (Methods). Figure 3b presents the data from 15 diamond probes (see Supplementary Note 3 for the full data set). We find *d*_2_ to show large variations between the probes and set-point parameters, with values ranging from 43–128 nm in the AM mode to 26–87 nm in the FM mode. We also find that FM feedback leads to a consistently lower stand-off and reduced spread in *d*_2_. A histogram analysis (Figure 3c) shows that the median stand-off value is reduced from 59.6 nm (AM) to 43.1 nm (FM), corresponding to a net reduction of 16.5 nm. This significant reduction has a strong impact on the image resolution and strength of the stray field signal (Figure 5), offering improved characterization in scanning magnetometry applications.

### Subsurface Depth of NV Centers

In a third step, we determine the subsurface depth, *d*_3_, of NV centers in the diamond probe by the technique of NV-NMR.^70,71^ Specifically, we use dynamical decoupling of the NV center to detect the magnetic noise from the ^1^H spins contained in the adsorbate layer on the diamond surface.^72,73^ Because the intensity of the ^1^H NMR signal quickly decreases with depth *d*_3_, the peak magnitude can be used as a reliable and quantitative depth gauge for near-surface NV centers.^56,74^

**Figure 4:**
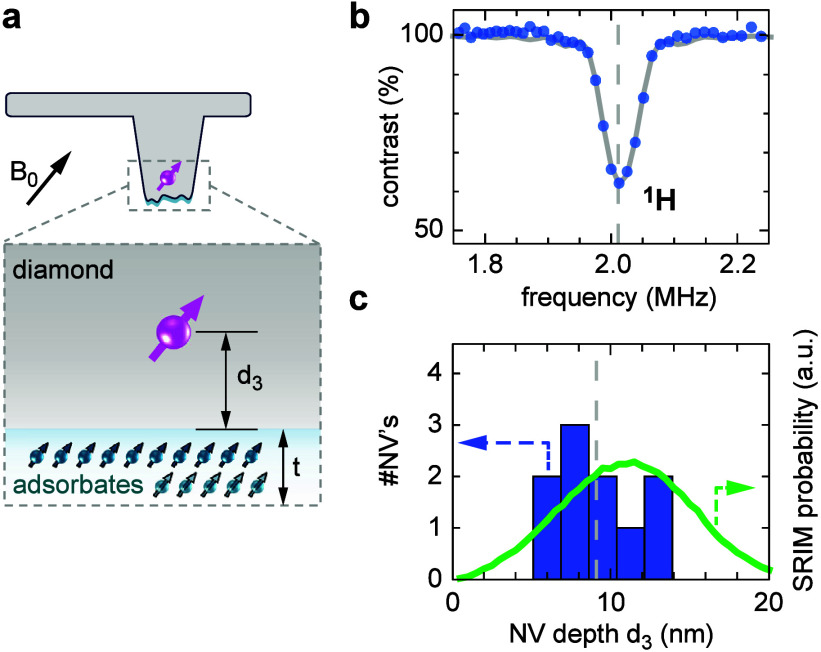
Subsurface depth of NV centers. (a) Calibration of the subsurface depth *d*_3_ by measuring the magnitude of the ^1^H NMR signal from the *t* ∼ 1 nm layer of surface adsorbates.^56^*B*_0_ is the bias field. (b) ^1^H NMR spectrum for NV7 recorded at *B*_0_ = 47.3 mT using dynamical decoupling spectroscopy.^75^ Dots are the experimental data, and the curve is a least-squares fit to the analytical model. The dashed line is the expected ^1^H resonance positions at this field. (c) Histogram of the measured *d*_3_ from 10 diamond probes. The gray dashed line is the median value. The green curve is the result of a SRIM simulation of the ^15^N^+^ ion distribution with depth *d*_3_.

Figure 4b shows the ^1^H NMR spectrum of a representative diamond probe together with the fit to an analytical model^73^ (see Supplementary Note 4 for further NMR spectra). The depth extracted from the fit is *d*_3_ = 5.7 ± 0.2 nm (Methods). A histogram collecting measurements from 10 diamond probes is shown in Figure 4c. We find that NV depths range from *d*_3_ = 5.1 to 13.9 nm with a median of 9.1 nm (dashed line). These values are in good agreement with a complementary Stopping and Range of Ions in Matter (SRIM) Monte Carlo simulation^77^ for the 7 keV ^15^N^+^ ions used in tip fabrication (10.8 ± 4.0 nm)^78^ (solid curve). From Figure 4c, we conclude that the subsurface depths for our scanning probes are consistent with the chosen implantation energy. In particular, we find no selection bias toward deeper NV centers, and the narrow distribution in *d*_3_ cannot explain the large variation in magnetic stand-off *d*_2_.

Table 1 concludes our stand-off analysis by presenting values for the distances *d*_1_, *d*_2_, and *d*_3_ for two scanning probes. A comparison between *d*_1_^AM^–*d*_1_^FM^ and *d*_2_^AM^–*d*_2_^FM^ shows that the reduction in the stand-off is roughly consistent between the mechanical and magnetic measurements, although the reduction is greater for the latter. This shows that our feedback control is reliable in adjusting the stand-off and does not lead to unexpected variation. A comparison with *d*_3_ also confirms that the subsurface NV depth is only a minor contribution to the magnetic stand-off *d*_2_.

**Table 1:** Mechanical Stand-off (*d*_1_), Magnetic Stand-off (*d*_2_), and Subsurface NV Depth (*d*_3_) for Two Diamond Probes^a^

	mechanical stand-off			magnetic stand-off			NV depth
diamond probe	*d*_1_^AM^, nm	*d*_1_^FM^, nm	*d*_1_^AM^–*d*_1_^FM^, nm	*d*_2_^AM^, nm	*d*_2_^FM^, nm	*d*_2_^AM^–*d*_2_^FM^, nm	*d*_3_, nm
NV2	2.5	–25.5	28.0	99.8	59.2	40.6	12.9
NV15	3.5	–2.5	6.0	53.8	43.1	10.7	5.1

a Set-point parameters are *A*_set_ = 75% and Δ*f*_res_ = 200 Hz (NV2) and *A*_set_ = 75% and Δ*f*_res_ = 135 Hz (NV15). Note that *d*_1_ + *d*_3_ ≠ *d*_2_ due to our definition of the *d*_1_ = 0 mechanical contact point (Figure 2b).

Table 1 also exemplifies that the magnetic stand-off varies greatly between probes, corroborating our findings from Figure 3. We hypothesize that the large variations are due to a combination of topographic features on the diamond tip (see AFM images in Figure 1c,d) and irregular meniscus formation^79^ as the tip moves into soft contact with the sample surface; in the future, these could be reduced by tip cleaning protocols^80^ and operation under a controlled atmosphere. FM feedback partially surmounts the mechanical barrier, placing the tip deep into the soft contact regime. Because the tuning fork has a high stiffness, a considerable force can be applied to the tip. This force will tend to level out soft features on both surfaces and reduce tip tilt if present. Below, we provide further evidence for this hypothesis by detecting the ^19^F NMR signal from a polytetrafluoroethylene (PTFE) film in soft contact.

### Magnetic Imaging with High Spatial Resolution

**Figure 5:**
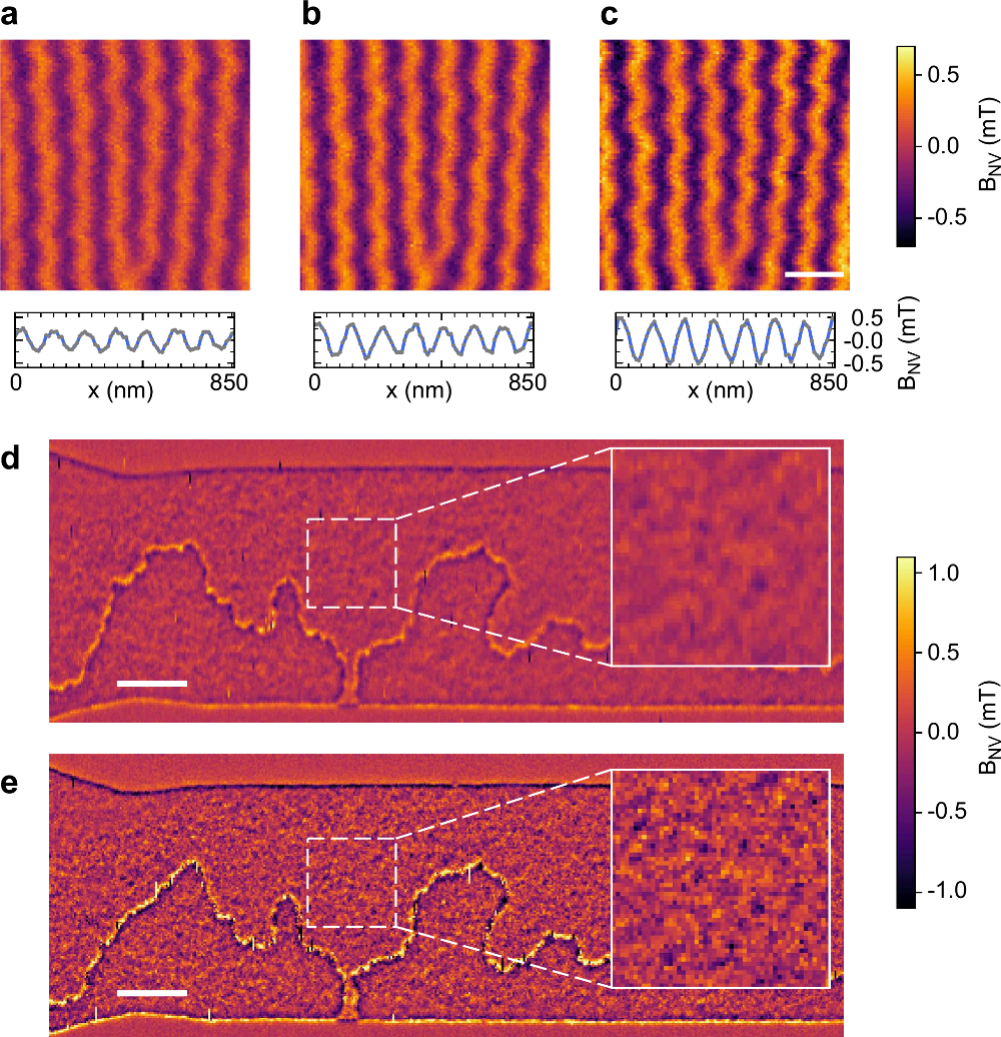
High-resolution magnetic field imaging of BiFeO_3_ and CoFeB. Quantitative stray field maps of a BiFeO_3_ thin film. (a) AM-AFM at 50% set point. (b) FM-AFM at Δ*f*_res_ = 65 Hz set point. (c) FM-AFM at Δ*f*_res_ = 155 Hz set point. The line cuts underneath the images show the periodic stray field oscillation of the BiFeO_3_ spin cycloid. Stray field amplitudes (peak-to-peak) are 0.47 ± 0.03 mT for part a, 0.67 ± 0.07 mT for part b, and 0.87 ± 0.05 mT for part c. Scale bar, 200 nm. (d,e) Quantitative stray field maps of a CoFeB synthetic antiferromagnetic racetrack measured in AM mode (d; 25% set point) and FM mode (e; Δ*f*_res_ = 110 Hz set point). The dashed-squared regions are shown at higher magnification as insets. Scale bars are 1 μm, and insets are 1.25 × 1.25 μm.

We now demonstrate that the lower stand-off available through FM feedback allows for a much improved spatial resolution in nanoscale imaging magnetometry applications. As our first example, we present images of the spin cycloid of bismuth ferrite (BiFeO_3_), an archetypal multiferroic material with noncollinear antiferromagnetic textures^22,51,52,81−83^ (see Methods and Supplementary Note 5 for further details). Parts a–c of Figure 5 show scanning NV images recorded in AM mode (panel a) and in FM mode with weak (panel b) and strong (panel c) frequency set points. The line cuts plotted underneath the images reveal that the peak-to-peak value of the stray field increases by 85% upon going from AM (panel a) to FM (panel c) feedback.

Because the stray field of a periodic magnetic structure decays as  with *d*_2_, where λ is one full spatial period, we can relate the signal increase to the change in the stand-off distance. Considering a period of λ = 119 nm (extracted using Fourier analysis of Figure 5c) and the 85% signal change, the reduction in the stand-off is approximately 12 nm. This value is consistent with the result obtained using inverse Fourier filtering (Supplementary Note 5). Overall, parts a–c of Figure 5 demonstrate that even a modest reduction in the stand-off distance can lead to large improvements in the magnetic signal.

Parts d and e of Figure 5 present a second example of comparative imaging using a fully compensated synthetic antiferromagnetic racetrack sample. The sample is composed of two 10.25-Å-thick layers of CoFeB separated by a Ru/Pt spacer, leading to OOP antiferromagnetic coupling (Methods). Clearly, the FM image (panel e) shows the device outline and domain walls within the racetrack in crisper detail compared to the AM image (panel d). The insets reveal that the root-mean-square (rms) values of the stray field increase from 0.13 mT (AM) to 0.30 mT (FM), corresponding to a signal rise by 130%. Separate fits to the stray field profiles at the edge of the racetrack (Supplementary Note 6) show that the magnetic stand-off is reduced from *d*_2_ = 43.6 ± 8.2 nm (AM) to *d*_2_ = 24.3 ± 4.6 nm (FM). The latter value is the smallest magnetic stand-off distance measured in this study.

### Detection of Meniscus Formation and Molecular Uptake

**Figure 6:**
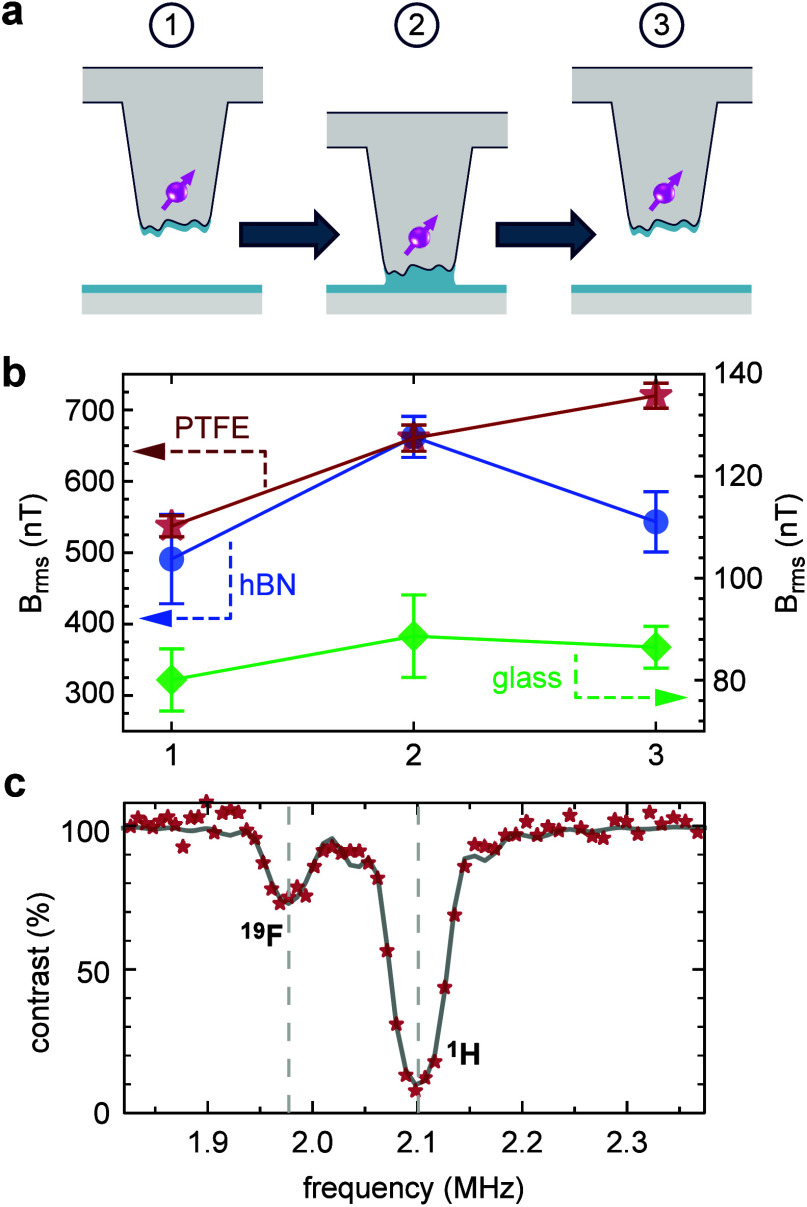
Investigation of capillary bridge formation and molecular adsorption using NV-NMR. (a) Experimental sequence: measurements are performed in free-space (1), soft contact (2), and after retraction to free space (3). (b) Magnitude of the ^1^H NMR peak (*B*_rms_) as a function of position 1–2–3. Data shown are for hBN (blue, acquired with NV15), PTFE (red, NV7), and glass substrate (green, NV16). Note that the data were taken with different tips (different *d*_3_), such that *B*_rms_ values are not directly comparable between substrates. (c) NMR spectrum taken in soft contact with a PTFE surface, revealing both ^19^F and ^1^H signals. The spectrum is acquired using NV7 and a bias field of 49.3 mT. Dashed lines are the expected resonance positions at this field. Fitting of the ^19^F peak (gray curve) yields *B*_rms_ = 295.6 ± 21.2 nT, corresponding to a NV-to-PTFE distance of *d*_2_ = 7.9 ± 0.4 nm (Methods).

Finally, in an attempt to reduce the stand-off even further, we investigate meniscus formation^79^ and molecular uptake in the soft contact regime (*d*_1_ < 0) using ^1^H and ^19^F NMR. As shown in Figure 6a, we approach the tip from free space (1) to soft contact (2) and then retract the tip to the original free-space position (3). At each position, we recorded the ^1^H NMR signal using NV-NMR (Figure 4). By calibrating the signal magnitude (*B*_rms_) in the initial free-space position, we can relate changes in the peak intensity during the approach–retract cycle to the amount of ^1^H-containing material within the detection volume of the NV center.^56^

Figure 6b shows soft-contact measurements performed over three representative substrates: hexagonal boron nitride (hBN) as an important capping layer in two-dimensional materials research, PTFE as an extremely hydro- and oleophobic material, and a glass microscopy slide (see Supplementary Note 4 for NMR spectra). For all substrates, we observe an increase in the ^1^H NMR peak as the tip approaches from position 1 to position 2. This reflects an increase in the thickness of the adsorbate layer, which we explain by meniscus formation. When the tip is retracted to position 3, however, different behaviors are observed for the three substrates: for hBN and glass, the ^1^H NMR signal decreases, albeit not to the level prior to approach. The decrease is stronger for hBN and almost absent for glass. A similar “residue effect” has been reported previously^84^ and has been ascribed to a local redistribution of adsorbate molecules. For the extremely hydrophobic PTFE, by contrast, the ^1^H NMR signal slightly increases because molecules preferentially adhere to the diamond surface, leading the tip to collect adsorbates from the PTFE surface.

For PTFE, we also measured the ^19^F NMR signal. Upon the tip approach (position 2), we observe a clear ^19^F NMR peak (Figure 6c), proving that the tip is in intimate contact with the sample. Converting the *B*_rms_ magnitude of the ^19^F NMR signal into a stand-off distance, we determine a value of *d*_2_ = 7.9 ± 0.4 nm, a value only marginally larger than the subsurface depth *d*_3_ = 5.7 ± 0.2 nm for this probe (Figure 4b). Together, our results demonstrate that in soft contact the gap between the probe and surface can be minimized and stand-off distances below 10 nm are achieved. The increase in *B*_rms_ after retraction of the tip (Figure 6b) also indicates that soft contact can lead to an increase in adsorbates. Measurements over repeated engage–retract cycles, however, show no increase in the ^1^H NMR intensity (Figure S6) and no increase in the magnetic stand-off (Figure S3), suggesting that the adsorbate uptake is a one-time effect.

## Conclusions

In summary, we present significant advances to the understanding of stand-off distances in SNVM microscopy, which are crucial to improving the spatial resolution and sensitivity of the technique. Starting with a detailed analysis of the tip approach curve, we show that FM feedback control of the tip position achieves a consistent improvement over AM feedback. The advance manifests both in improved tip approach (median stand-off of 43 nm for FM compared to 60 nm for AM modes) and more consistent stand-off values between different tips and experimental runs. The best-effort stand-off, measured by scanning across a magnetic step edge, is 24 nm. We demonstrate that even modest reductions in the stand-off distance can lead to dramatic improvements in the image quality and signal magnitude. These improvements are valuable not only to the ODMR-based imaging demonstrated here but also to alternating-current sensing^34,85,86^ and dynamic imaging modalities like scanning gradiometry.^87,88^ Finally, we explore the soft-contact regime, including capillary bridge formation and molecular uptake using NV-NMR, and show that sub-10-nm stand-off distances with SNVM can be reached. At this point, the subsurface NV depth *d*_3_ becomes relevant. Lowering the ion implantation energy during NV synthesis or using nitrogen-doped capping layers have demonstrated *d*_3_ < 3 nm.^56,73,89−91^ These results indicate that even lower stand-off distances, perhaps less than 5 nm, might be feasible.

Looking forward and in pursuit of a higher spatial resolution, the apex diameter of the diamond tip is a crucial parameter: in this study, diamond probes with rather large apex diameters of several hundred nanometers are used. While the diameter improves photon outcoupling,^92^ it also makes the tip more prone to topographic irregularities including residues from tip fabrication and particle pickup during scanning. In addition, large-diameter tips are more affected by meniscus formation and demand a more careful adjustment of the tip tilt. Advanced nanofabrication techniques should allow the engineering of diamond probes with substantially smaller end diameters, below 100 nm, without unduly compromising the photon yield.^45,93^ Further, by operating the SNVM in a controlled atmosphere or a high-vacuum environment, surface adsorbates could be reduced or entirely eliminated. Alternatively, passivation of the diamond surface using atomic layer deposition^94^ or suitable chemical groups^56^ might also reduce tip adhesion.

## Methods

### Scanning NV Microscope

The experiments are performed using a commercial scanning NV magnetometer (QSM, QZabre AG), which operates under ambient conditions. Scanning images are acquired by scanning the sample underneath the tip using a three-axis piezostage, while the tip remains stationary. The tuning-fork oscillator is actuated in shear mode to provide a force feedback needed for adjusting the tip–sample distance and performing AFM scanning.^95−97^ A top-side objective is used to both illuminate the diamond tip using a green diode laser (λ = 516 nm) and collect the resultant NV PL emission (band-pass filtered between λ ∼ 630 and 800 nm) using a single-photon-counting module. The optics are operated in a confocal configuration to suppress the luminescence background. Microwave pulses for manipulating the spin states of the NV center are generated by a short bond wire loop passing within 30 μm from the diamond tip. Either a permanent magnet or a vector electromagnet is employed for generating bias magnetic fields of up to 50 mT.

### AM and FM Feedback

The tuning-fork oscillator was controlled by using an Anfatec AFM controller. The tuning fork was driven electrically by applying a voltage between the electrodes patterned on the two prongs. To detect the tuning-fork oscillation, the current between the electrodes is measured by using a transimpedance amplifier. The Anfatec controller combines a digital lock-in amplifier, PLL, and PI feedback for controlling the amplitude and frequency set points. In AM feedback mode, the tuning fork is driven at the free-space resonance with a constant drive amplitude, and the tuning-fork-oscillation amplitude held at a constant set point (typically between 20 and 90% of the free-space amplitude) by feeding back on the *z* position. In FM feeedback mode, the tuning-fork oscillation is driven at resonance using the PLL, and the tuning-fork resonance frequency is held at a constant offset Δ*f*_res_ from the free-space frequency (typically between 5 and 200 Hz) by feeding back on the *z* position.

### Diamond Tip Fabrication

Diamond tips are fabricated from electronic-grade single-crystalline substrates with a {100} surface cut (Element6) using a series of lithography and etching steps.^45^ All tips characterized in this study are commercial scanning probes (QST, QZabre AG), except for the tips shown in Figure 1c,d, which are part of a larger pillar array.^45^ Count rates of NV centers range from 150 to 600 kCts/s, and the continuous-wave ODMR contrast varies between 12% and 26%.

### Magnetic Samples

*Co stripe*. The calibration sample for magnetic stand-off measurements^46,47^ is a roughly 2-μm-wide stripe of Pt(6 nm)/Co(1.6 nm)/Al(2 nm) with OOP anisotropy.^68^ The stripe is milled with Ar ions through a PMMA mask patterned by electron-beam lithography. The top Al layer is oxidized with a gentle oxygen plasma (power, 30 W) in an oxygen pressure of 10 mTorr to induce perpendicular magnetic anisotropy.^69^

*BiFeO*_3_. The BiFeO_3_ sample is grown by pulsed-laser deposition on SmScO_3_(110)_o_ (o denotes orthorhombic) substrates. The thickness of the BiFeO_3_ film is between 33 and 54 nm.^51^

*CoFeB*. The CoFeB synthetic antiferromagnetic racetrack is fabricated from a Ta(3 nm)/Pt(4 nm)/CoFeB(1.025 nm)/Ru(0.72 nm)/Pt(0.45 nm)/CoFeB(1.025 nm)/Ru(0.5 nm)/Ta(1.5 nm) thin-film stack, where numbers in parentheses represent nominal thicknesses. The films are deposited at room temperature by direct-current magnetron sputtering in an Ar pressure of 1 mTorr on high-resistivity Si substrates.

### Substrates Used for the Detection of Meniscus Formation

*PTFE*. Commercially available PTFE plates (APSOparts) were cleaned by sonication in acetone for 10 min, followed by isopropyl alcohol for 2 min, and then blow-dried using N_2_ gas.

*hBN*. The hBN substrate was prepared by exfoliation from hBN crystals (courtesy of K. Watanabe and T. Taniguchi) onto a silicon substrate chip with a 90-nm-thick silicon dioxide layer (dry chlorinated thermal oxide with forming gas anneal) using blue tape (Nitto ELP BT-150-E-CM), following the method described in ref (86).

*Glass*. Commercial glass coverslips (Knittel Glasbearbeitung) were used with no extra cleaning steps.

### Fitting of the Magnetic Stand-off

The stray field profile from a uniform, OOP magnetized stripe in the thin-film limit can be modeled by two antiparallel bound currents flowing at either edge of the stripe^46^
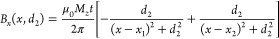
1
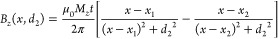
2
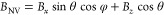
3where *M*_*z*_ is the OOP magnetization, *t* is the thickness of the ferromagnetic layer, *x*_1_ and *x*_2_ are the positions of stripe edges, and θ and φ are the polar and azimuth angles of the NV center in the laboratory (scanning) frame, respectively. By fitting of the measured stray field using eq 3, the magnetic stand-off distance *d*_2_ is determined. Note that the analytical model assumes a perfect OOP magnetization that is uniform across the film. This model may be oversimplified because the magnetization can deviate from the OOP alignment at the edges due to the demagnetizing field. The tilting will lead to an overestimation of *d*_2_. However, as long as the edge region where the magnetization deviates from OOP is much narrower than the stand-off distance, the effect is negligible.^47^

### NMR Depth Measurements

Surface adsorbates like water or hydrocarbons are naturally present on diamond under ambient conditions and have a typical thickness of 1–2 nm.^56,72,73^ This adsorbate layer has served as a reliable depth gauge in previous NV-NMR studies.^56,73,74,78^ We detected the ^1^H NMR signal through the reduction in NV spin coherence using dynamical decoupling spectroscopy. The exact measurement protocol is given in Supplementary Note 4. The magnitude of the coherence dip can be converted into a rms value *B*_rms_ of magnetic field noise generated by the ^1^H spins. *B*_rms_ is strongly dependent on the distance between the NV center and ^1^H layer and can thus be used to precisely determine *d*_3_. We use the analytical expression of refs (73) and (78):
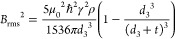
4where μ_0_ is the vacuum magnetic permeability, *ℏ* is the reduced Planck constant, γ = 2π × 42.57 MHz/T is the proton gyromagnetic ratio, ρ is the proton density, and *t* is the thickness of the surface adsorption layer. We assume values of ρ = 60 (nm)^−3^ typical for water or hydrocarbons and *t* = 1.3 nm from a previous study.^78^

## Data Availability

The data that support the plots within this paper and other findings of this study are available from the corresponding authors upon reasonable request.

## Supporting Information Available

AFM imaging of diamond probes, approach curves, magnetic stand-off measurements, NV depth measurements using ^1^H NMR, and imaging of BiFeO_3_ and CoFeB (PDF)

## References

[ref1] Rugar D.; Mamin H.; Guethner P.; Lambert S.; Stern J.; McFadyen I.; Yogi T. Magnetic Force Microscopy: General Principles and Application to Longitudinal Recording Media. J. Appl. Phys. 1990, 68, 1169–1183. 10.1063/1.346713.

[ref2] Hartmann U. Magnetic Force Microscopy. Annual review of materials science 1999, 29, 53–87. 10.1146/annurev.matsci.29.1.53.

[ref3] Marshall A.; Klein L.; Dodge J.; Ahn C.; Reiner J.; Mieville L.; Antagonazza L.; Kapitulnik A.; Geballe T.; Beasley M. Lorentz Transmission Electron Microscope Study of Ferromagnetic Domain Walls in SrRuO3: Statics, Dynamics, and Crystal Structure Correlation. Journal of applied physics 1999, 85, 4131–4140. 10.1063/1.370322.

[ref4] Yu X.; Onose Y.; Kanazawa N.; Park J. H.; Han J.; Matsui Y.; Nagaosa N.; Tokura Y. Real-Space Observation of a Two-Dimensional Skyrmion Crystal. Nature 2010, 465, 901–904. 10.1038/nature09124. 20559382

[ref5] Kirtley J. R.; Wikswo J. P. Scanning SQUID Microscopy. Annu. Rev. Mater. Sci. 1999, 29, 117–148. 10.1146/annurev.matsci.29.1.117.

[ref6] Choi Y.; Kemmer J.; Peng Y.; Thomson A.; Arora H.; Polski R.; Zhang Y.; Ren H.; Alicea J.; Refael G.; von Oppen F.; Watanabe K.; Taniguchi T.; Nadj-Perge S. Electronic Correlations in Twisted Bilayer Graphene Near the Magic Angle. Nat. Phys. 2019, 15, 1174–1180. 10.1038/s41567-019-0606-5.

[ref7] Nuckolls K. P.; Oh M.; Wong D.; Lian B.; Watanabe K.; Taniguchi T.; Bernevig B. A.; Yazdani A. Strongly Correlated Chern Insulators in Magic-Angle Twisted Bilayer Graphene. Nature 2020, 588, 610–615. 10.1038/s41586-020-3028-8. 33318688

[ref8] Zhang Y.; Polski R.; Lewandowski C.; Thomson A.; Peng Y.; Choi Y.; Kim H.; Watanabe K.; Taniguchi T.; Alicea J. others. Promotion of Superconductivity in Magic-Angle Graphene Multilayers. Science 2022, 377, 1538–1543. 10.1126/science.abn8585. 36173835

[ref9] Grzybowski M. J.; Wadley P.; Edmonds K. W.; Beardsley R.; Hills V.; Campion R. P.; Gallagher B. L.; Chauhan J. S.; Novak V.; Jungwirth T.; Maccherozzi F.; Dhesi S. S. Imaging current-induced switching of antiferromagnetic domains in cumnas. Phys. Rev. Lett. 2017, 118, 057701 10.1103/PhysRevLett.118.057701. 28211721

[ref10] Chernobrod B. M.; Berman G. P. Spin Microscope Based on Optically Detected Magnetic Resonance. J. Appl. Phys. 2005, 97, 014903 10.1063/1.1829373.

[ref11] Degen C. L. Scanning Magnetic Field Microscope With a Diamond Single-Spin Sensor. Appl. Phys. Lett. 2008, 92, 243111 10.1063/1.2943282.

[ref12] Balasubramanian G.; Chan I. Y.; Kolesov R.; Al-Hmoud M.; Tisler J.; Shin C.; Kim C.; Wojcik A.; Hemmer P. R.; Krueger A.; Hanke T.; Leitenstorfer A.; Bratschitsch R.; Jelezko F.; Wrachtrup J. Nanoscale Imaging Magnetometry With Diamond Spins Under Ambient Conditions. Nature 2008, 455, 648 10.1038/nature07278. 18833276

[ref13] Rondin L.; Tetienne J. P.; Rohart S.; Thiaville A.; Hingant T.; Spinicelli P.; Roch J. F.; Jacques V. Stray-Field Imaging of Magnetic Vortices With a Single Diamond Spin. Nat. Commun. 2013, 4, 2279 10.1038/ncomms3279. 23900221

[ref14] Tetienne J. P.; Hingant T.; Kim J.; Diez L. H.; Adam J. P.; Garcia K.; Roch J. F.; Rohart S.; Thiaville A.; Ravelosona D.; Jacques V. Nanoscale Imaging and Control of Domain-Wall Hopping With a Nitrogen-Vacancy Center Microscope. Science 2014, 344, 1366–1369. 10.1126/science.1250113. 24948732

[ref15] Tetienne J. P.; et al. The Nature of Domain Walls in Ultrathin Ferromagnets Revealed by Scanning Nanomagnetometry. Nat. Commun. 2015, 6, 6733 10.1038/ncomms7733. 25828294

[ref16] Dussaux A.; Schoenherr P.; Koumpouras K.; Chico J.; Chang K.; Lorenzelli L.; Kanazawa N.; Tokura Y.; Garst M.; Bergman A.; Degen C. L.; Meier D. Local Dynamics of Topological Magnetic Defects in the Itinerant Helimagnet FeGe. Nat. Commun. 2016, 7, 12430 10.1038/ncomms12430. 27535899 PMC4992142

[ref17] Appel P.; Shields B. J.; Kosub T.; Hedrich N.; Hubner R.; Fassbender J.; Makarov D.; Maletinsky P. Nanomagnetism of Magnetoelectric Granular Thin-Film Antiferromagnets. Nano Lett. 2019, 19, 1682–1687. 10.1021/acs.nanolett.8b04681. 30702895 PMC6422036

[ref18] Wornle M. S.; Welter P.; Kaspar Z.; Olejnik K.; Novak V.; Campion R. P.; Wadley P.; Jungwirth T.; Degen C. L.; Gambardella P. Current-Induced Fragmentation of Antiferromagnetic Domains. arXiv, 2019. https://arxiv.org/abs/1912.05287 (accessed 02-06-2025).

[ref19] Wornle M. S.; Welter P.; Giraldo M.; Lottermoser T.; Fiebig M.; Gambardella P.; Degen C. L. Coexistence of Bloch and Neél Walls in a Collinear Antiferromagnet. Phys. Rev. B 2021, 103, 094426 10.1103/PhysRevB.103.094426.

[ref20] Hedrich N.; Wagner K.; Pylypovskyi O. V.; Shields B. J.; Kosub T.; Sheka D. D.; Makarov D.; Maletinsky P. Nanoscale Mechanics of Antiferromagnetic Domain Walls. Nat. Phys. 2021, 17, 574 10.1038/s41567-020-01157-0.

[ref21] Finco A.; Haykal A.; Tanos R.; Fabre F.; Chouaieb S.; Akhtar W.; Robert-Philip I.; Legrand W.; Ajejas F.; Bouzehouane K.; Reyren N.; Devolder T.; Adam J.-P.; Kim J.-V.; Cros V.; Jacques V. Imaging Non-Collinear Antiferromagnetic Textures via Single Spin Relaxometry. Nat. Commun. 2021, 12, 767 10.1038/s41467-021-20995-x. 33536440 PMC7859235

[ref22] Gross I.; Akhtar W.; Garcia V.; Martinez L. J.; Chouaieb S.; Garcia K.; Carretero C.; Barthelemy A.; Appel P.; Maletinsky P.; Kim J.-V.; Chauleau J. Y.; Jaouen N.; Viret M.; Bibes M.; Fusil S.; Jacques V. others. Real-space Imaging of Non-Collinear Antiferromagnetic Order with a Single-Spin Magnetometer. Nature 2017, 549, 252–256. 10.1038/nature23656. 28905889

[ref23] Chauleau J.; et al. Electric and Antiferromagnetic Chiral Textures at Multiferroic Domain Walls. Nat. Mater. 2020, 19, 386–390. 10.1038/s41563-019-0516-z. 31685944

[ref24] Lorenzelli L. Development of a Scanning Nitrogen-Vacancy-Center Magnetometer for Variable Temperature Experiments. Ph.D. Thesis, ETH Zurich, Zurich, Switzerland, 2021.

[ref25] Thiel L.; Wang Z.; Tschudin M. A.; Rohner D.; Gutierrez-lezama I.; Ubrig N.; Gibertini M.; Giannini E.; Morpurgo A. F.; Maletinsky P. Probing Magnetism in 2D Materials at the Nanoscale with Single-Spin Microscopy. Science 2019, 364, 973 10.1126/science.aav6926. 31023891

[ref26] Sun Q.; Song T.; Anderson E.; Brunner A.; Forster J.; Shalomayeva T.; Taniguchi T.; Watanabe K.; Grafe J.; Stohr R.; Xu X.; Wrachtrup J. Magnetic Domains and Domain Wall Pinning in Atomically Thin CrBr_3_ Revealed by Nanoscale Imaging. Nat. Commun. 2021, 12, 1989 10.1038/s41467-021-22239-4. 33790290 PMC8012586

[ref27] Fabre F.; Finco A.; Purbawati A.; Hadj-Azzem A.; Rougemaille N.; Coraux J.; Philip I.; Jacques V. Characterization of Room-Temperature In-Plane Magnetization in Thin Flakes of CrTe_2_ With a Single-Spin Magnetometer. Phys. Rev. Materials 2021, 5, 034008 10.1103/PhysRevMaterials.5.034008.

[ref28] Dovzhenko Y.; Casola F.; Schlotter S.; Zhou T. X.; Buttner F.; Walsworth R. L.; Beach G. S. D.; Yacoby A. Magnetostatic Twists in Room-Temperature Skyrmions Explored by Nitrogen-Vacancy Center Spin Texture Reconstruction. Nat. Commun. 2018, 9, 2712 10.1038/s41467-018-05158-9. 30006532 PMC6045603

[ref29] Gross I.; Akhtar W.; Hrabec A.; Sampaio J.; Martinez L. J.; Chouaieb S.; Shields B. J.; Maletinsky P.; Thiaville A.; Rohart S.; Jacques V. Skyrmion Morphology in Ultrathin Magnetic Films. Phys. Rev. Materials 2018, 2, 024406 10.1103/PhysRevMaterials.2.024406.

[ref30] Jenkins A.; Pelliccione M.; Yu G.; Ma X.; Li X.; Wang K. L.; Jayich A. C. B. Single-Spin Sensing of Domain-Wall Structure and Dynamics in a Thin-Film Skyrmion Host. Phys. Rev. Materials 2019, 3, 083801 10.1103/PhysRevMaterials.3.083801.

[ref31] Thiel L.; Rohner D.; Ganzhorn M.; Appel P.; Neu E.; Muller B.; Kleiner R.; Koelle D.; Maletinsky P. Quantitative Nanoscale Vortex Imaging Using a Cryogenic Quantum Magnetometer. Nat. Nanotechnol. 2016, 11, 677 10.1038/nnano.2016.63. 27136133

[ref32] Pelliccione M.; Jenkins A.; Ovartchaiyapong P.; Reetz C.; Emmanouilidou E.; Ni N.; Bleszynski Jayich A. C. Scanned Probe Imaging of Nanoscale Magnetism at Cryogenic Temperatures. Nat. Nanotechnol. 2016, 11, 700–705. 10.1038/nnano.2016.68. 27136130

[ref33] Chang K.; Eichler A.; Rhensius J.; Lorenzelli L.; Degen C. L. Nanoscale Imaging of Current Density With a Single-Spin Magnetometer. Nano Lett. 2017, 17, 2367 10.1021/acs.nanolett.6b05304. 28329445

[ref34] Palm M. L.; Ding C.; Huxter W. S.; Taniguchi T.; Watanabe K.; Degen C. L. Observation of Current Whirlpools in Graphene at Room Temperature. Science 2024, 384, 465–469. 10.1126/science.adj2167. 38662845

[ref35] Schmid I.; Marioni M. A.; Kappenberger P.; Romer S.; Parlinska-wojtan M.; Hug H. J.; Hellwig O.; Carey M. J.; Fullerton E. E. Exchange Bias and Domain Evolution at 10 nm Scales. Phys. Rev. Lett. 2010, 105, 197201 10.1103/PhysRevLett.105.197201. 21231192

[ref36] Bode M.; Vedmedenko E.; Von Bergmann K.; Kubetzka A.; Ferriani P.; Heinze S.; Wiesendanger R. Atomic Spin Structure of Antiferromagnetic Domain Walls. Nature materials 2006, 5, 477–481. 10.1038/nmat1646. 16680147

[ref37] Zhang C.; Chuu C.-P.; Ren X.; Li M.-Y.; Li L.-J.; Jin C.; Chou M.-Y.; Shih C.-K. Interlayer Couplings, Moiré Patterns, and 2D Electronic Superlattices in MoS_2_/WSe_2_ Hetero-Bilayers. Sci. Adv. 2017, 3, e1601459 10.1126/sciadv.1601459. 28070558 PMC5218515

[ref38] Crommie M. F.; Lutz C. P.; Eigler D. M. Confinement of Electrons to Quantum Corrals on a Metal Surface. Science 1993, 262, 218–220. 10.1126/science.262.5131.218. 17841867

[ref39] Heller E.; Crommie M.; Lutz C.; Eigler D. Scattering and Absorption of Surface Electron Waves in Quantum Corrals. Nature 1994, 369, 464–466. 10.1038/369464a0.

[ref40] Tisler J.; Oeckinghaus T.; Stöhr R. J.; Kolesov R.; Reuter R.; Reinhard F.; Wrachtrup J. Single Defect Center Scanning Near-Field Optical Microscopy on Graphene. Nano Lett. 2013, 13, 3152–3156. 10.1021/nl401129m. 23795752

[ref42] Rondin L.; Tetienne J. P.; Hingant T.; Roch J. F.; Maletinsky P.; Jacques V. Magnetometry With Nitrogen-Vacancy Defects in Diamond. Rep. Prog. Phys. 2014, 77, 056503 10.1088/0034-4885/77/5/056503. 24801494

[ref43] Schirhagl R.; Chang K.; Loretz M.; Degen C. L. Nitrogen-Vacancy Centers in Diamond: Nanoscale Sensors for Physics and Biology. Annu. Rev. Phys. Chem. 2014, 65, 83 10.1146/annurev-physchem-040513-103659. 24274702

[ref44] Maletinsky P.; Hong S.; Grinolds M. S.; Hausmann B.; Lukin M. D.; Walsworth R. L.; Loncar M.; Yacoby A. A Robust Scanning Diamond Sensor for Nanoscale Imaging with Single Nitrogen-Vacancy Centres. Nat. Nanotechnol. 2012, 7, 320–324. 10.1038/nnano.2012.50. 22504708

[ref45] Zhu T.; Rhensius J.; Herb K.; Damle V.; Puebla-Hellmann G.; Degen C. L.; Janitz E. Multicone Diamond Waveguides for Nanoscale Quantum Sensing. Nano Lett. 2023, 23, 10110–10117. 10.1021/acs.nanolett.3c02120. 37934929

[ref46] Hingant T.; Tetienne J.-P.; Martínez L.; Garcia K.; Ravelosona D.; Roch J.-F.; Jacques V. Measuring the Magnetic Moment Density in Patterned Ultrathin Ferromagnets with Submicrometer Resolution. Physical Review Applied 2015, 4, 014003 10.1103/PhysRevApplied.4.014003.

[ref47] Tetienne J.-P.; Hingant T.; Martinez L.J.; Rohart S.; Thiaville A.; Diez L. H.; Garcia K; Adam J.-P.; Kim J.-V.; Roch J.-F.; Miron I.M.; Gaudin G.; Vila L.; Ocker B.; Ravelosona D.; Jacques V. The Nature of Domain Walls in Ultrathin Ferromagnets Revealed by Scanning Nanomagnetometry. Nat. Commun. 2015, 6, 1–6. 10.1038/ncomms7733. 25828294

[ref48] Gross I.; Martinez L. J.; Tetienne J.-P.; Hingant T.; Roch J.-F.; Garcia K.; Soucaille R.; Adam J. P.; Kim J.-V.; Rohart S.; Thiaville A.; Torrejon J.; Hayashi M.; Jacques V. Direct Measurement of Interfacial Dzyaloshinskii-Moriya Interaction in X— CoFeB— MgO Heterostructures with a Scanning NV Magnetometer (X= Ta, TaN, and W). Phys. Rev. B 2016, 94, 064413 10.1103/PhysRevB.94.064413.

[ref50] Rohner D.; Happacher J.; Reiser P.; Tschudin M.; Tallaire A.; Achard J.; Shields B.; Maletinsky P. Oriented, Single Crystal Diamond Tips for Nanoscale Scanning Probe Imaging of Out-of-Plane Magnetic Fields. Appl. Phys. Lett. 2019, 115, 192401 10.1063/1.5127101.

[ref51] Zhong H.; Finco A.; Fischer J.; Haykal A.; Bouzehouane K.; Carretero C.; Godel F.; Maletinsky P.; Munsch M.; Fusil S.; Jacques V.; Garcia V. others Quantitative Imaging of Exotic Antiferromagnetic Spin Cycloids in BiFeO_3_ Thin Films. Phys. Rev. Appl. 2022, 17, 044051 10.1103/PhysRevApplied.17.044051.

[ref52] Finco A.; Haykal A.; Fusil S.; Kumar P.; Dufour P.; Forget A.; Colson D.; Chauleau J.-Y.; Viret M.; Jaouen N.; Garcia V.; Jacques V. others Imaging Topological Defects in a Noncollinear Antiferromagnet. Phys. Rev. Lett. 2022, 128, 187201 10.1103/PhysRevLett.128.187201. 35594103

[ref53] Finco A.; Jacques V. Single Spin Magnetometry and Relaxometry Applied to Antiferromagnetic Materials. APL Mater. 2023, 11. 10.1063/5.0167480

[ref54] Pham V. T.; et al. Fast Current-Induced Skyrmion Motion in Synthetic Antiferromagnets. Science 2024, 384, 307–312. 10.1126/science.add5751. 38635712

[ref55] QZabre Ltd. https://qzabre.com (accessed 02-06-2025).

[ref56] Janitz E.; Herb K.; Volker L. A.; Huxter W. S.; Degen C. L.; Abendroth J. M. Diamond Surface Engineering for Molecular Sensing with Nitrogen-Vacancy Centers. Journal of Materials Chemistry C 2022, 10, 13533–13569. 10.1039/D2TC01258H. 36324301 PMC9521415

[ref57] Lo Y.; Huefner N. D.; Chan W. S.; Dryden P.; Hagenhoff B.; Beebe T. P. Organic and Inorganic Contamination on Commercial AFM Cantilevers. Langmuir 1999, 15, 6522–6526. 10.1021/la990371x.

[ref58] Parthasarathy S.; Joos M.; Hughes L. B.; Meynell S. A.; Morrison T. A.; Risner-Jamtgaard J.; Weld D. M.; Mukherjee K.; Jayich A. C. B. Role of Oxygen in Laser Induced Contamination at Diamond-Vacuum Interfaces. arXiv, 2024. https://arxiv.org/abs/2401.06942 (accessed 02-06-2025).

[ref59] Enachescu M.; Carpick R. W.; Ogletree D. F.; Salmeron M. The Role of Contaminants in the Variation of Adhesion Friction and Electrical Conduction Properties of Carbide-Coated Scanning Probe Tips and Pt(111) in Ultrahigh Vacuum. J. Appl. Phys. 2004, 95, 7694 10.1063/1.1738536.

[ref60] Hoppe S.; Ctistis G.; Paggel J. J.; Fumagalli P. Spectroscopy of the Shear Force Interaction in Scanning Near-Field Optical Microscopy. Ultramicroscopy 2005, 102, 221–226. 10.1016/j.ultramic.2004.10.002. 15639353

[ref61] Buchler B.; Kalkbrenner T.; Hettich C.; Sandoghdar V. Measuring the Quantum Efficiency of the Optical Emission of Single Radiating Dipoles Using a Scanning Mirror. Physical review letters 2005, 95, 063003 10.1103/PhysRevLett.95.063003. 16090945

[ref62] Israelsen N. M.; Kumar S.; Tawfieq M.; Neergaard-Nielsen J. S.; Huck A.; Andersen U. L. Increasing the Photon Collection Rate from a Single NV Center with a Silver Mirror. Journal of optics 2014, 16, 114017 10.1088/2040-8978/16/11/114017.

[ref63] Ernst S.; Irber D. M.; Waeber A. M.; Braunbeck G.; Reinhard F. A Planar Scanning Probe Microscope. ACS Photonics 2019, 6, 327–331. 10.1021/acsphotonics.8b01583.

[ref64] Drummond Roby M.; Wetsel G. Measurement of Elastic Force on a Scanned Probe Near a Solid Surface. Applied physics letters 1996, 69, 3689–3691. 10.1063/1.117190.

[ref65] Pfeiffer O.; Bennewitz R.; Baratoff A.; Meyer E.; Grütter P. Lateral-Force Measurements in Dynamic Force Microscopy. Phys. Rev. B 2002, 65, 161403 10.1103/PhysRevB.65.161403.

[ref66] Karrai K.; Tiemann I. Interfacial Shear Force Microscopy. Phys. Rev. B 2000, 62, 13174 10.1103/PhysRevB.62.13174.

[ref67] Göttlich H.; Stark R. W.; Pedarnig J. D.; Heckl W. M. Noncontact Scanning Force Microscopy Based on a Modified Tuning Fork Sensor. Rev. Sci. Instrum. 2000, 71, 3104–3107. 10.1063/1.1304881.

[ref68] Wörnle M. S. Nanoscale Scanning Diamond Magnetometry of Antiferromagnets. Ph.D. Thesis, ETH Zurich, Zurich, Switzerland, 2021.

[ref69] Luo Z.; Dao T. P.; Hrabec A.; Vijayakumar J.; Kleibert A.; Baumgartner M.; Kirk E.; Cui J.; Savchenko T.; Krishnaswamy G.; Heyderman L. J.; Gambardella P. Chirally Coupled Nanomagnets. Science 2019, 363, 1435–1439. 10.1126/science.aau7913. 30923219

[ref70] Mamin H. J.; Kim M.; Sherwood M. H.; Rettner C. T.; Ohno K.; Awschalom D. D.; Rugar D. Nanoscale Nuclear Magnetic Resonance with a Nitrogen-Vacancy Spin Sensor. Science 2013, 339, 557–560. 10.1126/science.1231540. 23372008

[ref71] Staudacher T.; Shi F.; Pezzagna S.; Meijer J.; Du J.; Meriles C. A.; Reinhard F.; Wrachtrup J. Nuclear Magnetic Resonance Spectroscopy on a (5-nanometer)^3^ Sample Volume. Science 2013, 339, 561–563. 10.1126/science.1231675. 23372009

[ref72] Degen C. L.; Poggio M.; Mamin H. J.; Rettner C. T.; Rugar D. Nanoscale Magnetic Resonance Imaging. Proc. Nat. Acad. Sci. U.S.A. 2009, 106, 1313 10.1073/pnas.0812068106. PMC2628306 19139397

[ref73] Loretz M.; Pezzagna S.; Meijer J.; Degen C. Nanoscale Nuclear Magnetic Resonance with a 1.9-nm-Deep Nitrogen-Vacancy Sensor. Appl. Phys. Lett. 2014, 104, 033102 10.1063/1.4862749.

[ref74] Pham L. M.; Devience S. J.; Casola F.; Lovchinsky I.; Sushkov A. O.; Bersin E.; Lee J.; Urbach E.; Cappellaro P.; Park H.; Yacoby A.; Lukin M.; Walsworth R. L. NMR Technique for Determining the Depth of Shallow Nitrogen-Vacancy Centers in Diamond. Phys. Rev. B 2016, 93, 045425 10.1103/PhysRevB.93.045425.

[ref75] Bylander J.; Gustavsson S.; Yan F.; Yoshihara F.; Harrabi K.; Fitch G.; Cory D. G.; Nakamura Y.; Tsai J. S.; Oliver W. D. Noise Spectroscopy Through Dynamical Decoupling with a Superconducting Flux Qubit. Nat. Phys. 2011, 7, 565–570. 10.1038/nphys1994.

[ref77] Ziegler J. F.; Biersack J. P. Treatise on Heavy-Ion Science: volume 6: Astrophysics, Chemistry and Condensed Matter; Springer, 1985; pp 93–129.

[ref78] Abendroth J. M.; Herb K.; Janitz E.; Zhu T.; Volker L. A.; Degen C. L. Single-Nitrogen-Vacancy NMR of Amine-Functionalized Diamond Surfaces. Nano Lett. 2022, 22, 7294–7303. 10.1021/acs.nanolett.2c00533. 36069765

[ref79] Weeks B. L.; Vaughn M. W.; Deyoreo J. J. Direct Imaging of Meniscus Formation in Atomic Force Microscopy Using Environmental Scanning Electron Microscopy. Langmuir 2005, 21, 8096–8098. 10.1021/la0512087. 16114907

[ref80] Gan Y.; Franks G. V. Cleaning AFM Colloidal Probes by Mechanically Scrubbing with Supersharp Brushes. Ultramicroscopy 2009, 109, 1061–1065. 10.1016/j.ultramic.2009.03.019. 19361930

[ref81] Chauleau J.-Y.; Chirac T.; Fusil S.; Garcia V.; Akhtar W.; Tranchida J.; Thibaudeau P.; Gross I.; Blouzon C.; Finco A.; Bibes M.; Dkhil B.; Khalyavin D. D.; Manuel P.; Jacques V.; Jaouen N.; Viret M. Electric and Antiferromagnetic Chiral Textures at Multiferroic Domain Walls. Nat. Mater. 2020, 19, 386–390. 10.1038/s41563-019-0516-z. 31685944

[ref82] Haykal A.; Fischer J.; Akhtar W.; Chauleau J.-Y.; Sando D.; Finco A.; Godel F.; Birkholzer Y. A.; Carretero C.; Jaouen N.; Bibes M.; Viret M.; Fusil S.; Jacques V.; Garcia V. Antiferromagnetic Textures in BiFeO_3_ Controlled by Strain and Electric Field. Nat. Commun. 2020, 11, 1–7. 10.1038/s41467-020-15501-8. 32249777 PMC7136242

[ref83] Dufour P.; Abdelsamie A.; Fischer J.; Finco A.; Haykal A.; Sarott M. F.; Varotto S.; Carretero C.; Collin S.; Godel F.; Jaouen N.; Viret M.; Trassin M.; Bouzehouane K.; Jacques V.; Chauleau J.-Y.; Fusil S.; Garcia V. Onset of Multiferroicity in Prototypical Single-Spin Cycloid BiFeO_3_ Thin Films. Nano Lett. 2023, 23, 9073–9079. 10.1021/acs.nanolett.3c02875. 37737821

[ref84] Rugar D.; Mamin H.; Sherwood M.; Kim M.; Rettner C. T.; Ohno K.; Awschalom D. D. Proton Magnetic Resonance Imaging Using a Nitrogen-Vacancy Spin Sensor. Nat. Nanotechnol. 2015, 10, 120–124. 10.1038/nnano.2014.288. 25531089

[ref85] Ku M. J. H.; et al. Imaging Viscous Flow of the Dirac Fluid in Graphene. Nature 2020, 583, 537–541. 10.1038/s41586-020-2507-2. 32699401

[ref86] Palm M. L.; Huxter W. S.; Welter P.; Ernst S.; Scheidegger P. J.; Diesch S.; Chang K.; Rickhaus P.; Taniguchi T.; Watanabe K.; Ensslin K.; Degen C. L. Imaging of Submicroampere Currents in Bilayer Graphene Using a Scanning Diamond Magnetometer. Phys. Rev. Appl. 2022, 17. 10.1103/PhysRevApplied.17.054008

[ref87] Huxter W. S.; Palm M. L.; Davis M. L.; Welter P.; Lambert C. H.; Trassin M.; Degen C. L. Scanning Gradiometry with a Single Spin Quantum Magnetometer. Nat. Commun. 2022, 13, 3761 10.1038/s41467-022-31454-6. 35768430 PMC9243102

[ref88] Huxter W. S.; Sarott M. F.; Trassin M.; Degen C. L. Imaging Ferroelectric Domains with a Single-Spin Scanning Quantum Sensor. Nat. Phys. 2023, 19, 644 10.1038/s41567-022-01921-4. 37205126 PMC10185469

[ref89] Sangtawesin S.; et al. Origins of Diamond Surface Noise Probed by Correlating Single-Spin Measurements with Surface Spectroscopy. Phys. Rev. X 2019, 9, 031052 10.1103/PhysRevX.9.031052.

[ref90] Ohashi K.; Rosskopf T.; Watanabe H.; Loretz M.; Tao Y.; Hauert R.; Tomizawa S.; Ishikawa T.; Ishi-hayase J.; Shikata S.; Degen C. L.; Itoh K. M. Negatively Charged Nitrogen-Vacancy Centers in a 5 nm thin 12C Diamond Film. Nano Lett. 2013, 13, 4733–4738. 10.1021/nl402286v. 24020334

[ref91] Myers B. A.; Das A.; Dartiailh M. C.; Ohno K.; Awschalom D. D.; Bleszynski Jayich A. C. Probing Surface Noise with Depth-Calibrated Spins in Diamond. Phys. Rev. Lett. 2014, 113, 027602 10.1103/PhysRevLett.113.027602. 25062234

[ref92] Momenzadeh S. A.; Stohr R. J.; de Oliveira F. F.; Brunner A.; Denisenko A.; Yang S.; Reinhard F.; Wrachtrup J. Nanoengineered Diamond Waveguide as a Robust Bright Platform for Nanomagnetometry Using Shallow Nitrogen Vacancy Centers. Nano Lett. 2015, 15, 165–169. 10.1021/nl503326t. 25438091

[ref93] Hedrich N.; Rohner D.; Batzer M.; Maletinsky P.; Shields B. J. Parabolic Diamond Scanning Probes for Single-Spin Magnetic Field Imaging. Physical Review Applied 2020, 14, 064007 10.1103/PhysRevApplied.14.064007.

[ref94] Jones J. C.; Delegan N.; Heremans F. J.; Martinson A. B. Nucleation Dependence of Atomic Layer Deposition on Diamond Surface Termination. Carbon 2023, 213, 118276 10.1016/j.carbon.2023.118276.

[ref95] Edwards H.; Taylor L.; Duncan W.; Melmed A. J. Fast, High-Resolution Atomic Force Microscopy Using a Quartz Tuning Fork as Actuator and Sensor. Journal of applied physics 1997, 82, 980–984. 10.1063/1.365936.

[ref96] Giessibl F. J. High-speed Force Sensor for Force Microscopy and Profilometry Utilizing a Quartz Tuning Fork. Applied physics letters 1998, 73, 3956–3958. 10.1063/1.122948.

[ref97] Ruiter A.; Van Der Werf K.; Veerman J.; Garcia-Parajo M.; Rensen W.; Van Hulst N. Tuning Fork Shear-Force Feedback. Ultramicroscopy 1998, 71, 149–157. 10.1016/S0304-3991(97)00111-3. 9566344

