# Supplementary material for: Minimizing Sensor-Sample Distances in Scanning Nitrogen-Vacancy Magnetometry
Source: ACS Nano. 2025 Feb 21;19(8):8255–65. doi: 10.1021/acsnano.4c18460 (PMC11887488; doi:10.1021/acsnano.4c18460)
Supplement: Supplementary file 1 — nn4c18460_si_001.pdf [file nn4c18460_si_001.pdf]

Supplementary Information: Minimizing sensor-sample distances in scanning nitrogen-vacancy magnetometry

Zhewen Xu[1,2], Marius Palm[1], William Huxter[1], Konstantin Herb[1], John M. Abendroth[1], Karim Bouzehouane[3], Olivier Boulle[4], Mihai S. Gabor[5], Joseba Urrestarazu Larranaga[4], Andrea Morales[2], Jan Rhensius[2], Gabriel Puebla-Hellmann[2], and Christian L. Degen[1,6]

[1]*Department of Physics, ETH Zürich, Otto Stern Weg 1, 8093 Zürich, Switzerland.* [2]*QZabre AG, Neubrunnenstr. 50, 8050 Zürich, Switzerland.* [3]*Unité Mixte de Physique, CNRS, Thales, Université Paris-Saclay, 91767 Palaiseau, France.* [4]*Université Grenoble Alpes, CNRS, CEA, SPINTEC, 38054 Grenoble, France.* [5]*Technical University of Cluj-Napoca, Memorandumului 28, Cluj-Napoca 400347, Romania.* [6]*Quantum Center, ETH Zürich, 8093 Zürich, Switzerland.*

(Dated: February 5, 2025)

SUPPLEMENTARY NOTE 1. AFM IMAGES OF DIAMOND PROBES

We performed the AFM measurements on two different stationary tip arrays, labeled array A and array B. The root-mean-square (rms) values ($z_{\rm rms}$) and the valley-to-peak heights ($z_{\rm vp}$) are extracted from the AFM images similar to those shown in Fig. 1c,d in the main text. Table S1 collects $z_{\rm rms}$ and $z_{\rm vp}$ values from fifteen diamond pillars.

The $z_{\rm rms}$ of the diamond tips in array A are remarkably low, with a median value of 0.7 nm. This indicates a highly smooth surface. In contrast, tip array B exhibits a higher median $z_{\rm rms} \sim 3.6$ nm.

Despite the generally smooth surfaces indicated by the rms values, we observed protruding features on the surfaces of both diamond tip arrays. These features are characterized by the $z_{\rm vp}$ values. The $z_{\rm vp}$ values are more relevant than the $z_{\rm rms}$ values as they add to the mechanical stand-off distance d_1. The median $z_{\rm vp}$ are 5.5 nm for array A and 18.5 nm for array B. We hypothesize that these features originate from two primary sources:

- *Mask residues:* During the fabrication process, residues from the mask material (Al) may remain on the diamond surface. These residues are challenging to completely eliminate, even though the tip array is washed by tri-acid solution (1:1:1 H_2SO_4:HNO_3:$HClO_4$ by volume).
- *Dust particles:* Dust from the ambient environment can accumulate on the diamond tips, contributing to the observed surface features. These particles can be difficult to control and may lead to contamination.

	NV A1	NV A2	NV A3	NV A4	NV A5	NV A6	NV A7	NV A8	NV A9
$z_{\rm rms}$ (nm)	4.7 nm	9.8 nm	0.7 nm	0.7 nm	0.6 nm	0.7 nm	0.4 nm	0.8 nm	0.7 nm
$z_{\rm vp}$ (nm)	28.0 nm	37.5 nm	5.3 nm	10.7 nm	4.8 nm	4.8 nm	3.0 nm	5.5 nm	7.2 nm
	NV B1	NV B2	NV B3	NV B4	NV B5	NV B6			
$z_{\rm rms}$ (nm)	4.5 nm	4.2 nm	3.9 nm	2.0 nm	2.2 nm	3.3 nm			
$z_{\rm vp}$ (nm)	50.0 nm	19.0 nm	18.0 nm	17.0 nm	15.0 nm	26.0 nm			

Table S1. Root-mean-square ($z_{\rm rms}$) and valley-to-peak ($z_{\rm vp}$) roughness for two diamond tip arrays, including tip array A (nine NVs) and tip array B (six NVs).

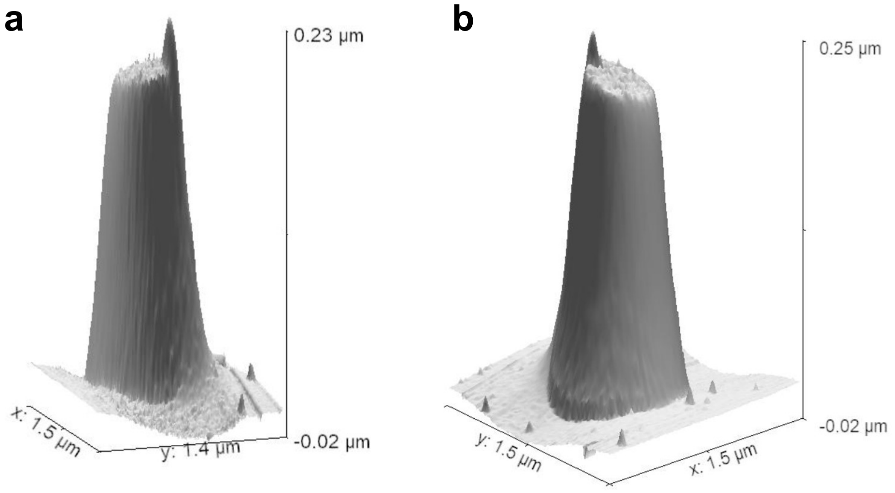

Figure S1. Reverse AFM images from two scanning tips (**a,b**). Note that the tip end surface is intentionally tilted by 10° and that only the top $\sim 0.25\ \mu$m of the 5-μm-high tip are reproduced.

In addition, we performed reverse AFM imaging of two scanning NV tips before use, see Fig. S1. Reverse AFM images were taken by scanning the NV tip over a calibration grid with sharp needles. Both tips presented protruding features with approximate heights of 25 to 30 nm. In summary, although the diamond tips present smooth surfaces with low rms roughness, they have a propensity for harboring particles on the tens of nanometer scale that add to the mechanical stand-off distance d_1.

SUPPLEMENTARY NOTE 2. FURTHER APPROACH CURVES

Fig. S2 shows approach curves from NV2, NV6, NV9, and NV15, complementing the approach curve for NV14 shown in the main text. Although the approach curves are quantitatively different, they all show the same qualitative features:

1. As the tip is approached towards the surface, the amplitude A_{res} decreases before the resonance frequency Δf_{res} increases. This behavior indicates conservative tip-sample interactions occur in a more closer tip-sample separation regime than dissipative tip-sample interactions.

2. The amplitude A_{res} presents a local minimum separating the non-contact and soft-contact regimes. The minimum can be used to define a contact point ($d_1 = 0$) for all tips.

3. In soft contact, A_{res} presents a non-monotonic variation including a local maximum, while Δf_{res} presents a monotonic increase. Therefore, FM feedback can be implemented in soft contact while AM feedback is unstable. The behavior of A_{res} and Δf_{res} aligns with observations in other lateral-oscillation-based scanning probe techniques, such as scanning near-field optical microscopy (SNOM) [1, 2] and shear-force microscopy [3–5].

4. The decrease of the PL signal extends beyond the lowest point of the oscillation amplitude A_{res}. This indicates that d_1 can still be lowered in soft contact mode.

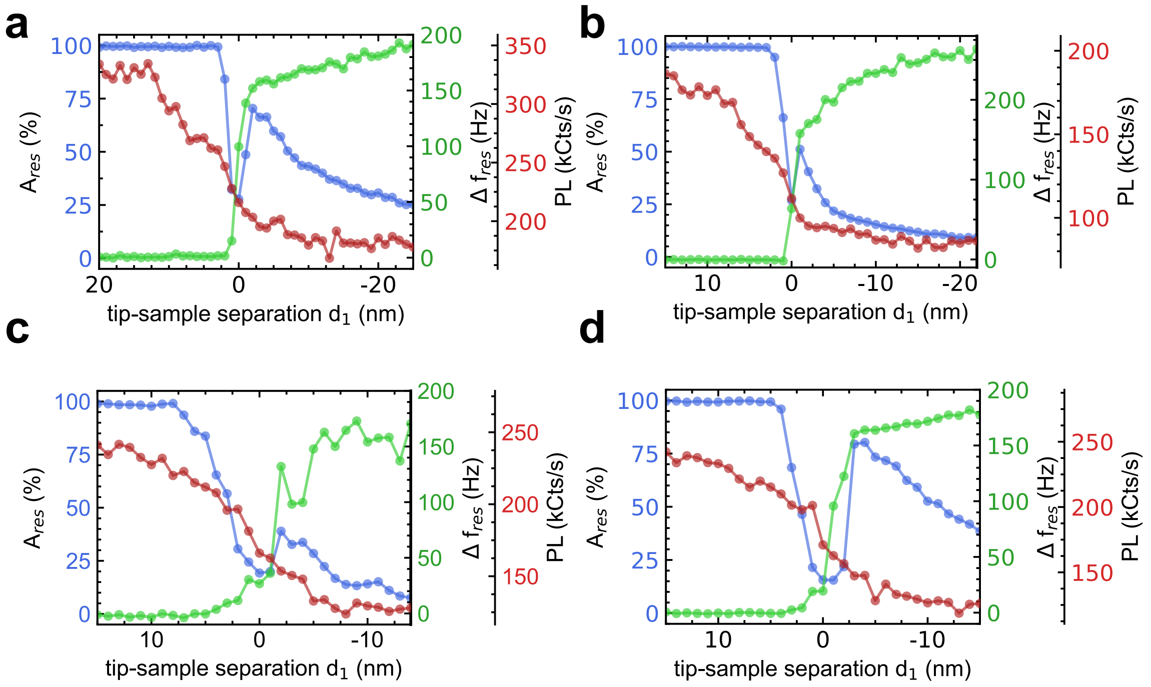

Figure S2. Approach curves plotting A_{res} (blue), Δf_{res} (green) and the PL intensity (red) as a function of d_1. The contact point $d_1 = 0$ is defined by the minimum in A_{res}. From **a-d**, NV2, NV6, NV9 and NV15.

SUPPLEMENTARY NOTE 3. MAGNETIC STAND-OFF MEASUREMENTS

Table S2 provides the set-point parameters and fitted magnetic stand-off distances d_2 for the dataset presented in Fig. 3b in the main text.

probe	magnetic stand-off distance d_2 (nm)		
NV1	126.6 ± 1.8 nm (A_{set} = 85 %)	128.0 ± 0.4 nm (A_{set} = 75 %)	110.6 ± 3.6 nm (A_{set} = 55 %)
	111.4 ± 2.9 nm (A_{set} = 30 %)	117.2 ± 2.9 nm (A_{set} = 20 %)	
	87.0 ± 3.7 nm (Δf_{res} = 80 Hz)	71.0 ± 0.8 nm (Δf_{res} = 110 Hz)	56.3 ± 3.4 nm (Δf_{res} = 125 Hz)
NV2	99.8 ± 2.0 nm (A_{set} = 75 %)	91.5 ± 2.7 nm (A_{set} = 50 %)	96.9 ± 0.9 nm (A_{set} = 20 %)
	87.1 ± 2.3 nm (Δf_{res} = 150 Hz)	59.2 ± 0.3 nm (Δf_{res} = 200 Hz)	
NV3	85.1 ± 0.6 nm (A_{set} = 75 %)	83.1 ± 0.4 nm (A_{set} = 45 %)	71.2 ± 2.0 nm (A_{set} = 20 %)
	73.4 ± 4.4 nm (Δf_{res} = 8 Hz)	47.2 ± 2.4 nm (Δf_{res} = 50 Hz)	42.5 ± 2.2 nm (Δf_{res} = 105 Hz)
NV4	80.2 ± 0.6 nm (A_{set} = 70 %)		
	59.8 ± 0.7 nm (Δf_{res} = 5 Hz)	55.1 ± 0.6 nm (Δf_{res} = 10 Hz)	
NV5	73.8 ± 1.4 nm (A_{set} = 45 %)	54.8 ± 0.5 nm (A_{set} = 20 %)	
	48.1 ± 0.4 nm (Δf_{res} = 73 Hz)	43.1 ± 0.8 nm (Δf_{res} = 145 Hz)	
NV6	67.3 ± 4.7 nm (A_{set} = 75 %)		
	33.2 ± 0.3 nm (Δf_{res} = 70 Hz)	30.2 ± 1.1 nm (Δf_{res} = 100 Hz)	
NV7	66.5 ± 2.5 nm (A_{set} = 75 %)	58.3 ± 1.5 nm (A_{set} = 66 %)	52.6 ± 2.2 nm (A_{set} = 50 %)
	50.9 ± 3.0 nm (A_{set} = 25 %)		
	25.7 ± 1.4 nm (Δf_{res} = 5 Hz)		
NV8	65.1 ± 1.0 nm (A_{set} = 75 %)	56.7 ± 1.0 nm (A_{set} = 55 %)	56.7 ± 3.0 nm (A_{set} = 35 %)
	54.4 ± 4.1 nm (A_{set} = 20 %)		
	55.4 ± 1.0 nm (Δf_{res} = 15 Hz)	52.8 ± 4.5 nm (Δf_{res} = 45 Hz)	
NV9	63.9 ± 0.3 nm (A_{set} = 75 %)	62.7 ± 1.9 nm (A_{set} = 50 %)	57.8 ± 0.4 nm (A_{set} = 25 %)
	52.7 ± 2.9 nm (Δf_{res} = 25 Hz)	47.1 ± 2.5 nm (Δf_{res} = 50 Hz)	41.5 ± 4.6 nm (Δf_{res} = 100 Hz)
NV10	63.1 ± 1.1 nm (A_{set} = 85 %)	52.3 ± 0.8 nm (A_{set} = 35 %)	
	41.8 ± 1.1 nm (Δf_{res} = 14 Hz)	36.3 ± 1.3 nm (Δf_{res} = 132 Hz)	
NV11	59.6 ± 1.0 nm (A_{set} = 85 %)	50.3 ± 0.6 nm (A_{set} = 75 %)	49.9 ± 3.3 nm (A_{set} = 50 %)
	40.3 ± 1.4 nm (Δf_{res} = 60 Hz)	36.6 ± 2.3 nm (Δf_{res} = 185 Hz)	34.9 ± 3.3 nm (Δf_{res} = 195 Hz)

NV12	59.4 ± 2.0 nm (A_{set} = 30 %)		
	49.9 ± 2.2 nm (Δf_{res} = 80 Hz)	48.6 ± 3.3 nm (Δf_{res} = 120 Hz)	
NV13	55.8 ± 0.9 nm (A_{set} = 66 %)		
	38.5 ± 1.3 nm (Δf_{res} = 5 Hz)	37.6 ± 1.3 nm (Δf_{res} = 7 Hz)	34.7 ± 0.6 nm (Δf_{res} = 8 Hz)
NV14	55.9 ± 0.4 nm (A_{set} = 70 %)	55.6 ± 0.6 nm (A_{set} = 65 %)	53.2 ± 1.5 nm (A_{set} = 50 %)
	43.7 ± 1.0 nm (Δf_{res} = 20 Hz)	37.1 ± 1.9 nm (Δf_{res} = 24 Hz)	33.8 ± 0.3 nm (Δf_{res} = 26 Hz)
	34.9 ± 0.8 nm (Δf_{res} = 29 Hz)	28.5 ± 5.5 nm (Δf_{res} = 165 Hz)	
NV15	53.8 ± 1.9 nm (A_{set} = 75 %)	52.5 ± 2.3 nm (A_{set} = 30 %)	43.3 ± 3.0 nm (A_{set} = 20 %)
	50.9 ± 3.0 nm (Δf_{res} = 50 Hz)	40.7 ± 0.6 nm (Δf_{res} = 70 Hz)	43.1 ± 0.3 nm (Δf_{res} = 135 Hz)
	40.2 ± 0.4 nm (Δf_{res} = 200 Hz)		

Table S2: Magnetic stand-off data (d_2).

Fig. S3 shows repeated measurements of the magnetic stand-off for tip NV2, demonstrating that in general, no significant increase in the magnetic stand-off is observed with repeated use.

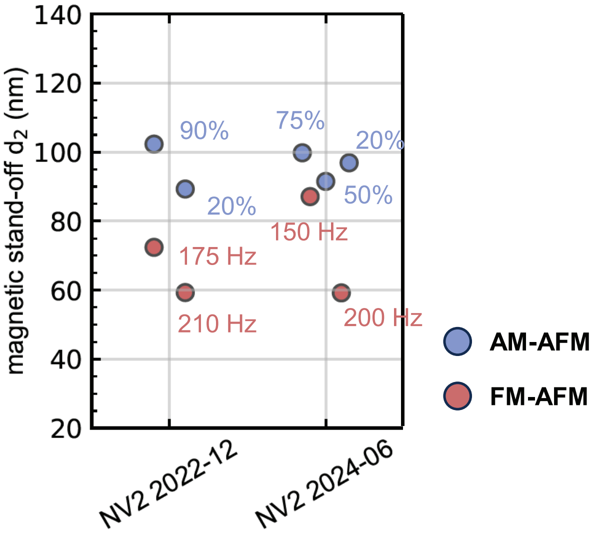

Figure S3. **Repeated measurements of the magnetic stand-off for tip NV2.** Nine stand-off measurements were performed (5x AM-AFM, 4x FM-AFM) at two different dates roughly 1.5 years apart. Hz and percentage number indicate set-point values.

SUPPLEMENTARY NOTE 4. DYNAMICAL DECOUPLING PROTOCOL AND ^{1}H NMR SPECTRA

We investigate the sub-surface depth d_3 using the technique of NV-NMR spectroscopy. This method measures the magnitude of the magnetic fluctuations (B_{rms}) from the statistically-polarized ^{1}H spins [6] from molecular adsorbates on the diamond surface [7]. The fluctuations are measured via the decoherence of the NV centers under a Carr-Purcell-Meiboom-Gill-type dynamic decoupling sequence [8, 9].

Fig. S4a illustrates the microwave and laser pulse protocol. Initially, the NV center is optically polarized into the $m_S = 0$ state. Subsequently, a $\pi/2$ pulse prepares it into a superposition state of $m_S = 0$ and $m_S = -1$. A sequence of N equidistant π pulses is then applied to dynamically decouple the NV center from the environmental noise. A final $\pi/2$ pulse is employed to project the superposition state back to the $\hat{S}_z$ basis of the spin-1 operators, which is then read out by a laser pulse. By adjusting the delay τ between the π

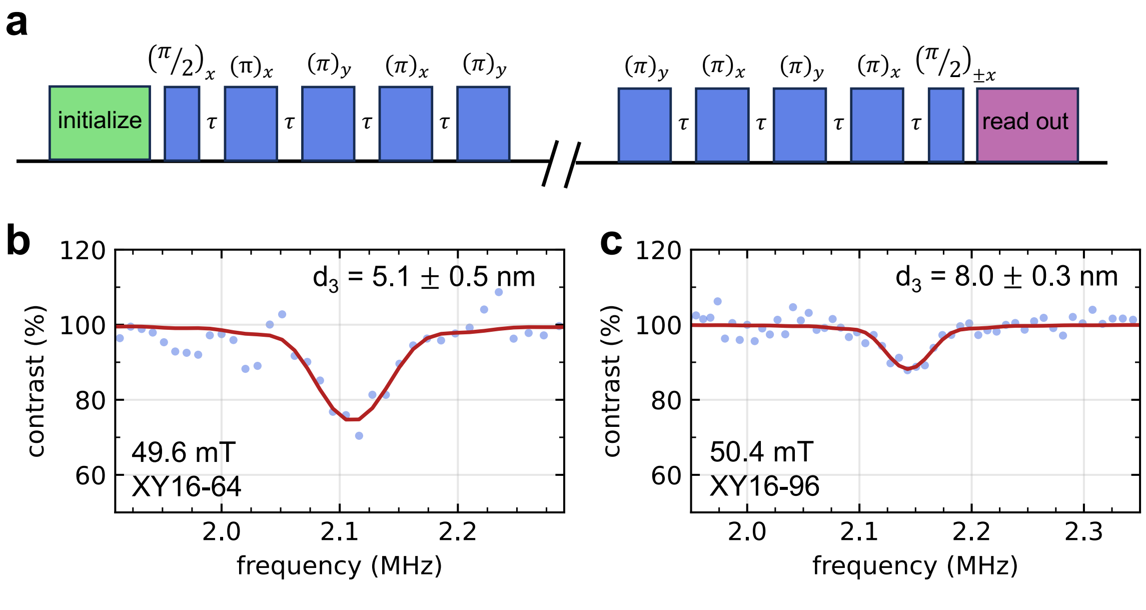

Figure S4. **XY-k protocol and NV-NMR spectra. a.** Dynamical decoupling spectroscopy sequence for detecting nuclear spin signals. **b** and **c.** Two spectra recorded from two different diamond probes listed in Fig. 4c of the main text. Dots represent experimental data and the curve is a least-squares fit to the analytical model. External bias field, XY16-N sequence and fitted d_3 are indicated in the figure.

pulses to half periodicity of the Larmor precession of examined nuclear spins, the magnetic fluctuation, quantified as a root mean square value B_{rms}, can be detected [10, 11]. The sub-surface depth, d_3, of NV centers, which correlates with the B_{rms}, is then extracted from an analytic expression (see Methods and Refs. [10, 11]).

Figs. S4b and c show ^{1}H NMR spectra of surface adsorbates obtained from NV15 and NV17, in addition to the spectrum of NV7 shown in the main text. NV15 (Fig. S4b) exhibits higher contrast with fewer π pulses, which corresponds to a shallower depth. Noted that all the diamond probes used for NV-NMR measurements are made from isotopically purified (>99.99% ^{12}C) diamond.

Fig. S5 shows the ^{1}H NMR spectra from which the B_{rms} values in Fig. 6b in the main text were extracted.

Fig. S6 shows repeated measurements of the proton-NMR signal intensity for tip NV15, indicating that the tip does not accumulate material over time.

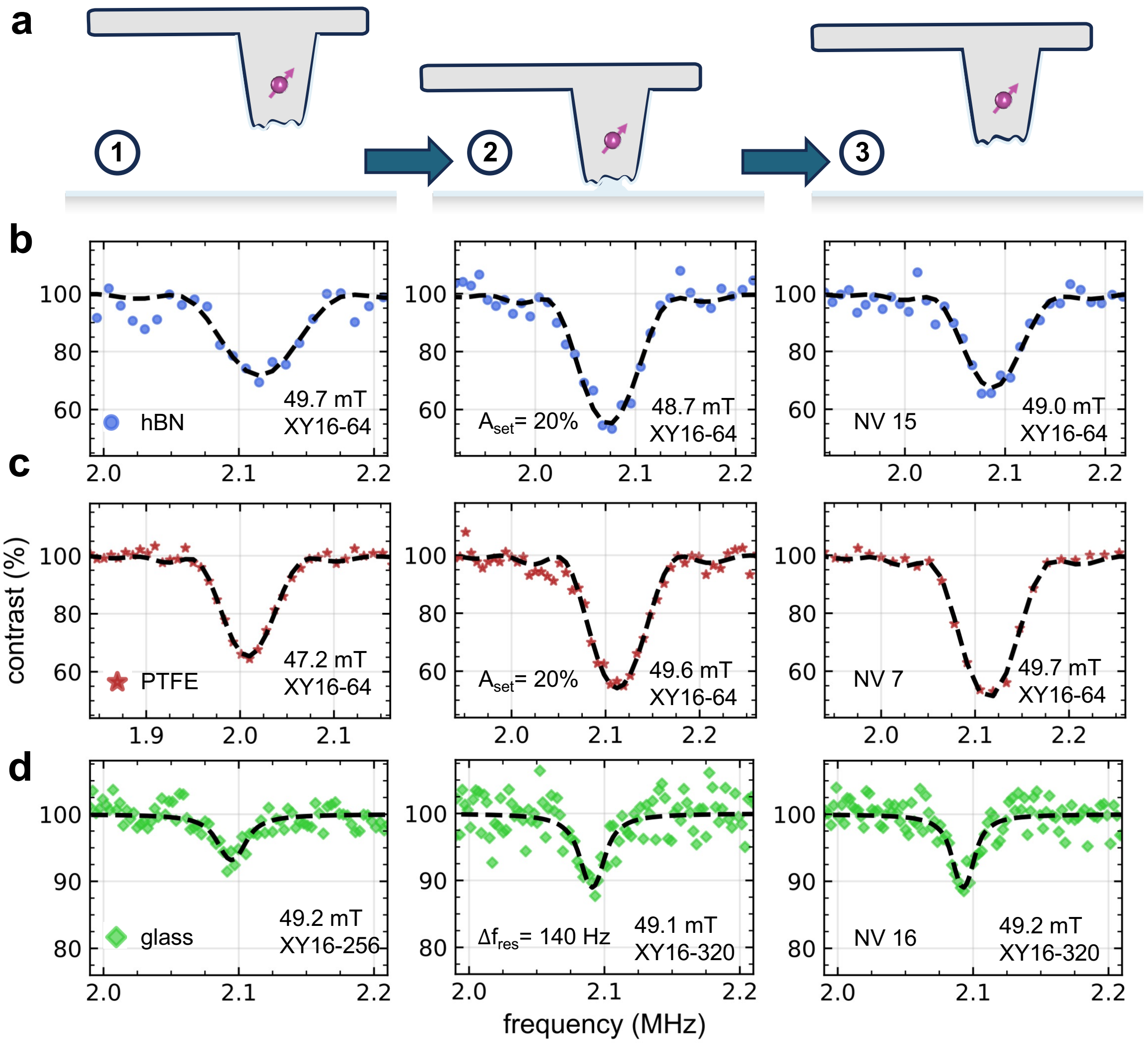

Figure S5. ^{1}H NMR spectra for to the capillary bridge measurement shown in Fig. 6b in the main text. **a.** Measurement sequence: (1) free space, (2) soft contact, (3) free space. **b-d.** ^{1}H NMR spectra taken with the indicated XY16-N pulse sequence, bias field and (if applicable) set-point parameter. Horizontal axis shows the detection frequency set by the dynamical decoupling sequence. Black dashed lines are fits to the data. The hBN data was taken with NV15, the PTFE data was taken with NV7, and the glass data was taken with NV16.

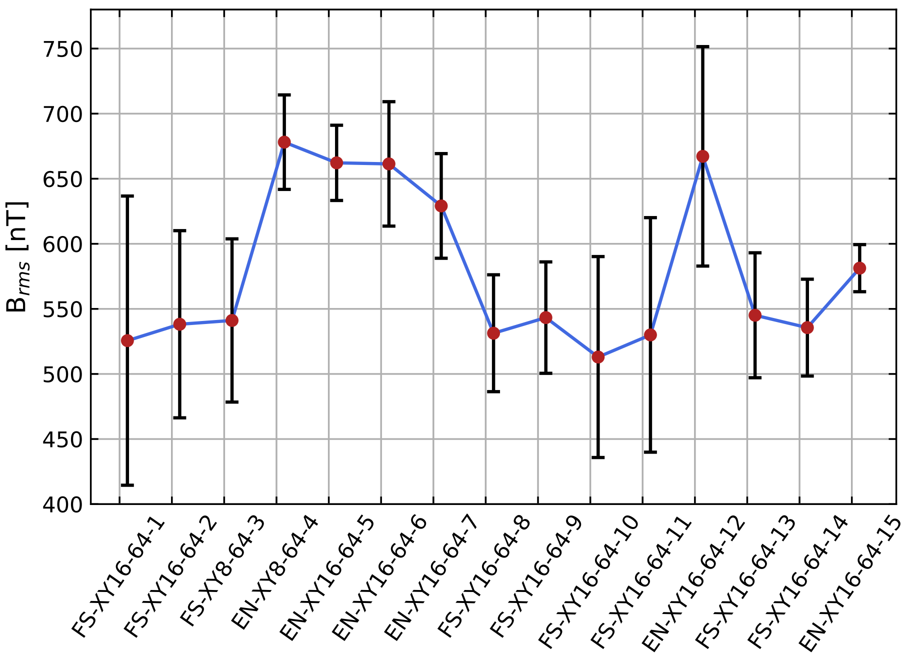

Figure S6. Repeated measurements of the proton-NMR signal intensity for tip NV15, given in units of rms magnetic field. “FS” and “EN” indicate that a measurement was performed in free-space and engaged (*i.e.*, soft contact) tip position, respectively. (Numbers following XY… identify the pulse protocol). Clearly, the FS-EN cycling is reversible and no increase in signal in FS position is observed with use, indicating that the tip does not accumulate material over time.

SUPPLEMENTARY NOTE 5. IMAGING OF $BiFeO_3$

Fig. S7a-c plots the two-dimensional (2D) FFT of the stray field maps of Figs. 5a-c in the main text. Two propagation directions of the spin cycloid are indentified, given by the k_1' and k_2' vectors in Fourier space. The line cuts along these two vectors are plotted underneath the 2D FFT images. By fitting the peaks with Lorentzian functions, the peak-to-peak amplitudes of the spin cycloid stray field are determined as 0.47 ± 0.03 mT (panel d), 0.67 ± 0.07 mT (panel e), and 0.87 ± 0.05 mT (panel f). The two vectors form an angle of $105.8 \pm 0.5°$, confirming the type II nature of the spin cycloid [12, 13].

Fig. S8 shows a second method for quantitatively determining the difference in stand-off height between the AM and FM images of $BiFeO_3$, complementing the method based on the $e^{-2\pi d_2/\lambda}$ decay of the stray field oscillation used in the main text. The procedure is based on vertically translating the FM in Fourier space until it matches the AM image. The detailed procedure is as follows:

- *Step 1:* The magnetic field measured in FM feedback (B_{FM}, Fig. S8d) is transformed into Fourier space ($\tilde{B}_{\mathrm{FM}}$) using a two-dimensional (xy) Fourier transform. The relative

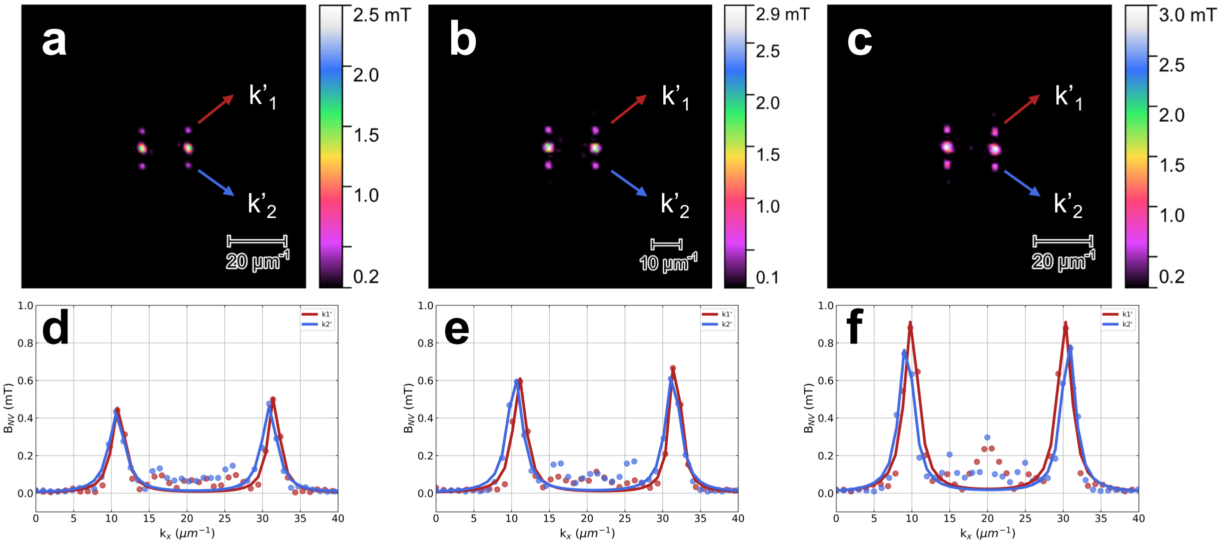

Figure S7. **Fourier analysis of $BiFeO_3$ images. a-c.** 2D FFT images of stray field maps shown in Figs. 5a-c. **d-f.** Line profiles (dots) underneath the images are the line profiles in Fourier space along the $BiFeO_3$ cycloid spin propagation directions k_1' (red) and k_2' (blue). Solid lines represent the fit results using Lorentzian functions.

height Δz is adjusted from 1 nm to 100 nm in 1-nm increments by multiplying $\tilde{B}_{\mathrm{FM}}$ with $e^{-k\Delta z}$, where $k = \sqrt{k_x^2 + k_y^2}$ is the reciprocal wave vector. Then, the resulting 100 images are transformed back to real space. Each image B'_{FM} is subtracted from the AM stray field image B_{AM} (Fig. S8c) pixel by pixel. The differences are squared and summed providing the cost function C. A preliminary Δz_1 is found by the minimum in C.

- *Step 2:* To compensate for xy drift between AM and FM images, using the preliminary Δz_1 determined in Step 1, the FM image is shifted in positive x direction by one pixel. The differences between the shifted B''_{FM} and B_{AM} are squared and summed. This procedure is repeated symmetrically for shifts up to five pixels along the x and y direction, respectively. The squared and summed differences C between the shifted B''_{FM} and B_{AM} are then plotted to find the new minimum, as shown in Fig. S8a.

- *Step 3:* Using the pixel shift determined in Step 3, which gives rises to the minimum C, Step 1 is repeated to obtain the relative height between the B_{AM} and B_{FM} images. Fig. S8b shows that the minimum for this dataset is at $\Delta z = 12\,\mathrm{nm}$.

- *Step 4:* Using the relative height $\Delta z = 12\,\mathrm{nm}$ determined in Step 3, the upward transformed $B'_{\mathrm{FM}}(\Delta z = 12\,\mathrm{nm})$ is presented in Fig. S8e. The absolute differences between B_{AM} and $B'_{\mathrm{FM}}(\Delta z = 12\,\mathrm{nm})$ are shown in Fig. S8f.

The Fourier translation procedure yields the same result ($\Delta z = 12\,\mathrm{nm}$) as the estimation based on the oscillation amplitude discussed in the main text, thus confirming our estimations.

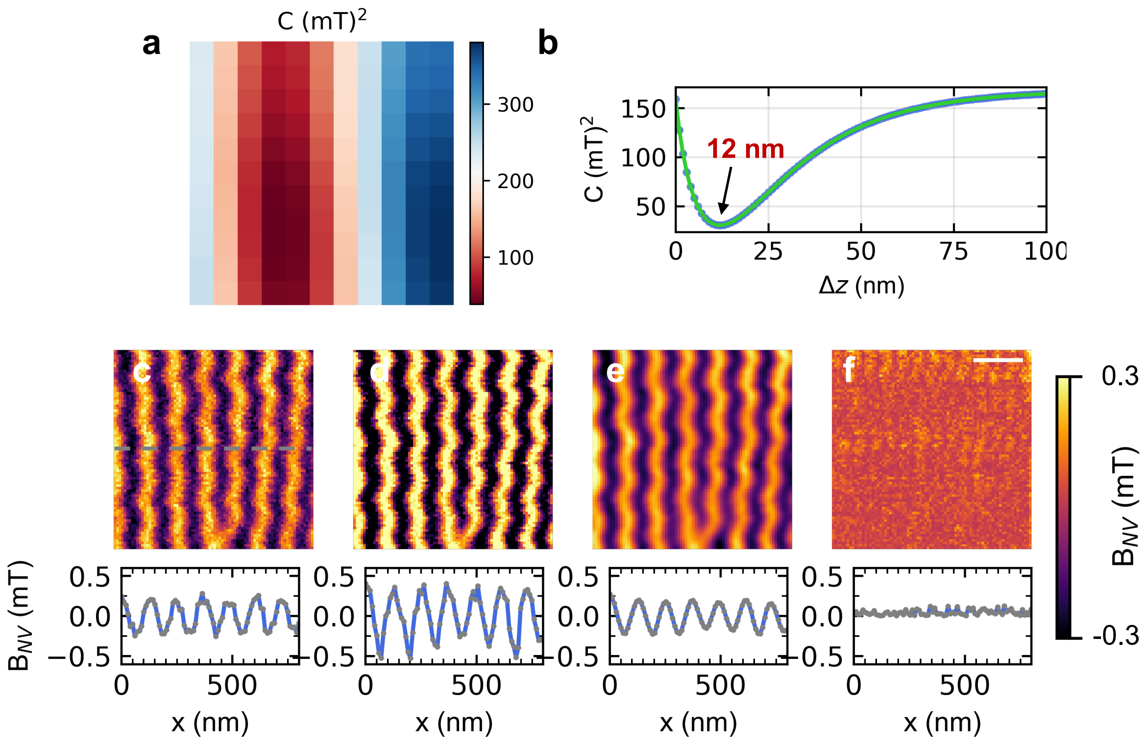

Figure S8. **Relative height between the AM and FM $BiFeO_3$ images. a.** The sum of the squared differences C between the shifted FM and AM images at a pre-determined relative distance Δz_1. x and y axes are pixel shifts. **b.** The sum of the squared differences between FM and AM images at different relative height. **c,d.** Stray field images B_{AM} and B_{FM} recorded using AM-AFM at a 50% set-point and FM-AFM at $\Delta f_{\mathrm{res}} = 155\,\mathrm{Hz}$ set-point, respectively (dataset from Fig. 5a,c in the main text). **e.** Vertically translated FM image B'_{FM} using a $\Delta z = 12\,\mathrm{nm}$ shift. **f.** Absolute difference between B_{AM} and B'_{FM} images. The scale bar is 200 nm. The line cuts (dot line in **c**) underneath the images (**c** to **f**) show the stray field from $BiFeO_3$.

SUPPLEMENTARY NOTE 6. IMAGING OF CoFeB

The CoFeB racetrack was also used to fit the stand-off distance d_2 by scanning over the racetrack edge. The analytical model for the step edge of a synthetic antiferromagnet (SAF) is given by

$$B_x(x, d_2) = \frac{\mu_0 M_z t}{2\pi}\left(-\frac{d_2}{(x-x_0)^2+d_2^2}\right) - \frac{\mu_0 M_z t}{2\pi}\left(-\frac{d_2'}{(x-x_0)^2+d_2'^2}\right), \tag{S1}$$

$$B_z(x, d_2) = \frac{\mu_0 M_z t}{2\pi}\left(\frac{x-x_0}{(x-x_0)^2+d_2^2}\right) - \frac{\mu_0 M_z t}{2\pi}\left(\frac{x-x_0}{(x-x_0)^2+d_2'^2}\right), \tag{S2}$$

$$B_{\mathrm{NV}} = B_x \sin\theta\cos\varphi + B_z\cos\theta, \tag{S3}$$

where M_z is the OOP magnetization, $t = 10.25\,\text{Å}$ is the thickness of one CoFeB layer, d_2 is the magnetic stand-off, $d_2' = d_2 + t + d_s$ (where $d_s = 11.7\,\text{Å}$ is the non-magnetic spacer between the two CoFeB layers), x_0 is the position of the step edge, and (θ,φ) is the projection axis of the NV center. Fifty line scans were fitted for each (AM, FM) image. The results are plotted in Fig. S9. The median stand-off values for the fits are $d_2 = 43.6 \pm 8.2\,\mathrm{nm}$ for the AM image and $d_2 = 24.3 \pm 4.6\,\mathrm{nm}$ for the FM image, respectively.

Further, Fig. S10 presents an analysis of the relative stand-off heights of AM and FM images using the Fourier translation method discussed with $BiFeO_3$.

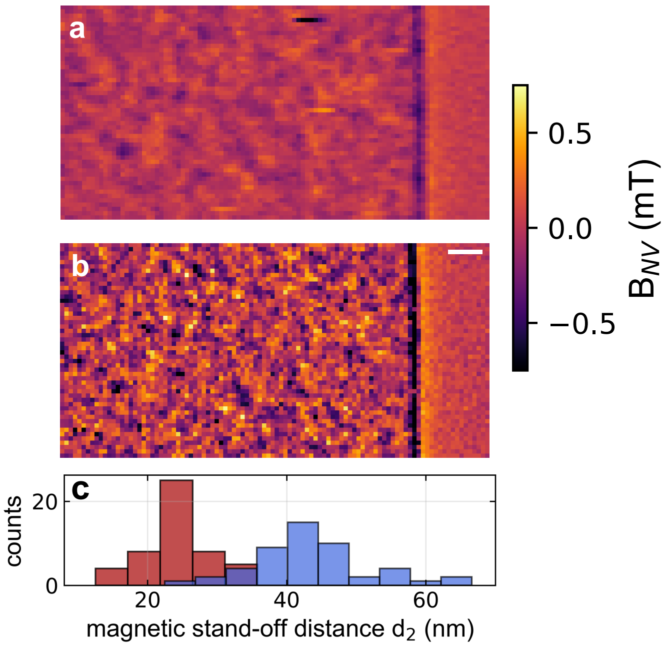

Figure S9. **Stand-off distances d_2 measured at the edge of the CoFeB racetrack a,b.** Magnetic field maps recorded AM-AFM at 25% set-point and FM-AFM at $\Delta f_{\mathrm{res}} = 110\,\mathrm{Hz}$ set-point, respectively. The scale bar is 200 nm. **c,d.** Histograms of d_2 obtained by fitting 50 linecuts from the AM (blue) and FM (red) images, respectively.

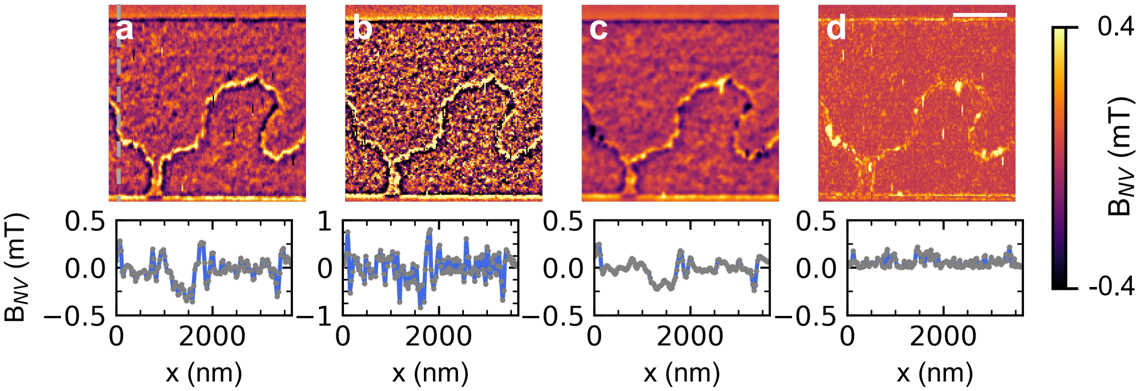

Figure S10. **Relative height between the AM and FM images for CoFeB. a,b.** Magnetic field images B_{AM} and B_{FM} recorded using AM-AFM at 25% set-point and FM-AFM at $\Delta f_{\mathrm{res}} = 110\,\mathrm{Hz}$ set-point, respectively. **c.** Vertically translated image B'_{FM} using a $\Delta z = 21\,\mathrm{nm}$ shift. **d.** Absolute difference between B_{AM} and B'_{FM} images. The scale bar is 1 μm. The line cuts (dot line in **a**) underneath the images show the stray field from CoFeB.

[1] H. Göttlich, R. W. Stark, J. D. Pedarnig, and W. M. Heckl, Noncontact scanning force microscopy based on a modified tuning fork sensor, Review of Scientific Instruments **71**, 3104 (2000).

[2] M.-P. Bernal, F. Marquis-Weible, P.-Y. Boillat, and P. Lambelet, Theoretical and experimental study of the forces between different snom probes and chemically treated afm cantilevers, Proceedings of the IEEE **88**, 1460 (2000).

[3] A. Ruiter, K. Van Der Werf, J. Veerman, M. Garcia-Parajo, W. Rensen, and N. Van Hulst, Tuning fork shear-force feedback, Ultramicroscopy **71**, 149 (1998).

[4] K. Karrai and I. Tiemann, Interfacial shear force microscopy, Physical Review B **62**, 13174 (2000).

[5] O. Pfeiffer, R. Bennewitz, A. Baratoff, E. Meyer, and P. Grütter, Lateral-force measurements in dynamic force microscopy, Physical review B **65**, 161403 (2002).

[6] C. L. Degen, M. Poggio, H. J. Mamin, and D. Rugar, Role of spin noise in the detection of small ensembles of nuclear spins, Phys. Rev. Lett. **99**, 250601 (2007).

[7] F. Maier, M. Riedel, B. Mantel, J. Ristein, and L. Ley, Origin of surface conductivity in diamond, Physical review letters **85**, 3472 (2000).

[8] H. Y. Carr and E. M. Purcell, Effects of diffusion on free precession in nuclear magnetic resonance experiments, Physical review **94**, 630 (1954).

[9] S. Meiboom and D. Gill, Modified spin-echo method for measuring nuclear relaxation times, Review of scientific instruments **29**, 688 (1958).

[10] M. Loretz, S. Pezzagna, J. Meijer, and C. Degen, Nanoscale nuclear magnetic resonance with a 1.9-nm-deep nitrogen-vacancy sensor, Applied Physics Letters **104**, 033102 (2014).

[11] L. M. Pham, S. J. Devience, F. Casola, I. Lovchinsky, A. O. Sushkov, E. Bersin, J. Lee, E. Urbach, P. Cappellaro, H. Park, A. Yacoby, M. Lukin, and R. L. Walsworth, NMR technique for determining the depth of shallow nitrogen-vacancy centers in diamond, Phys. Rev. B **93**, 045425 (2016).

[12] A. Haykal, J. Fischer, W. Akhtar, J.-Y. Chauleau, D. Sando, A. Finco, F. Godel, Y. Birkhölzer, C. Carrétéro, N. Jaouen, *et al.*, Antiferromagnetic textures in bifeo3 controlled by strain and electric field, Nature communications **11**, 1 (2020).

[13] H. Zhong, A. Finco, J. Fischer, A. Haykal, K. Bouzehouane, C. Carrétéro, F. Godel, P. Maletinsky, M. Munsch, S. Fusil, *et al.*, Quantitative imaging of exotic antiferromagnetic spin cycloids in bi fe o 3 thin films, Physical Review Applied **17**, 044051 (2022).
